# Lipoprotein proteome profile: novel insight into hyperlipidemia

**DOI:** 10.1002/ctm2.361

**Published:** 2021-04-08

**Authors:** Miao Lin, Menglin Li, Hao Zheng, Haidan Sun, Jinlan Zhang

**Affiliations:** ^1^ State Key Laboratory of Bioactive Substances and Functions of Natural Medicines Institute of Materia Medica Chinese Academy of Medical Sciences and Peking Union Medical College Beijing P.R. China; ^2^ Core facility of instrument Institute of Basic Medical Sciences Chinese Academy of Medical Sciences School of Basic Medicine, Peking Union Medical College Beijing China

Dear Editor,

Abnormal lipoprotein metabolism is related to hyperlipidemia^1^ and cardiovascular disease.^2^ Here, abnormal proteins of three different density lipoproteins were identified in hyperlipidemic golden hamsters by label‐free and targeted proteomics for the first time, including 13 new proteins. Functional analysis of differential proteins provided new insights into the relationship between lipoprotein components and hyperlipidemia. Comparing with previous studies,^3^, ^4^, ^5^ we achieve a better coverage and accuracy in lipoprotein proteins, providing a largest lipoprotein database to date.

Most studies focused on proteome study of one or two lipoproteins.^6^, ^7^ No simultaneous analysis of different lipoprotein classes was performed to assess their functional specificity and compare their association with hyperlipidemia. Golden hamster is a usual animal model for hyperlipidemia in preclinical studies,^8^ but its lipoproteome study is insufficient. Via label‐free proteomics, there are 129, 69, and 89 proteins identified in very‐low‐density lipoprotein (VLDL), low‐density lipoprotein (LDL), and high‐density lipoprotein (HDL) of golden hamster plasma, respectively (Figure 1A and Table S1), becoming the most comprehensive profiling of lipoproteins. Among them, 43, 18, and 9 new proteins were validated in that lipoprotein using parallel reaction monitoring (PRM) targeted proteomics (Figures 1B–1C and Table S2).

**FIGURE 1:**
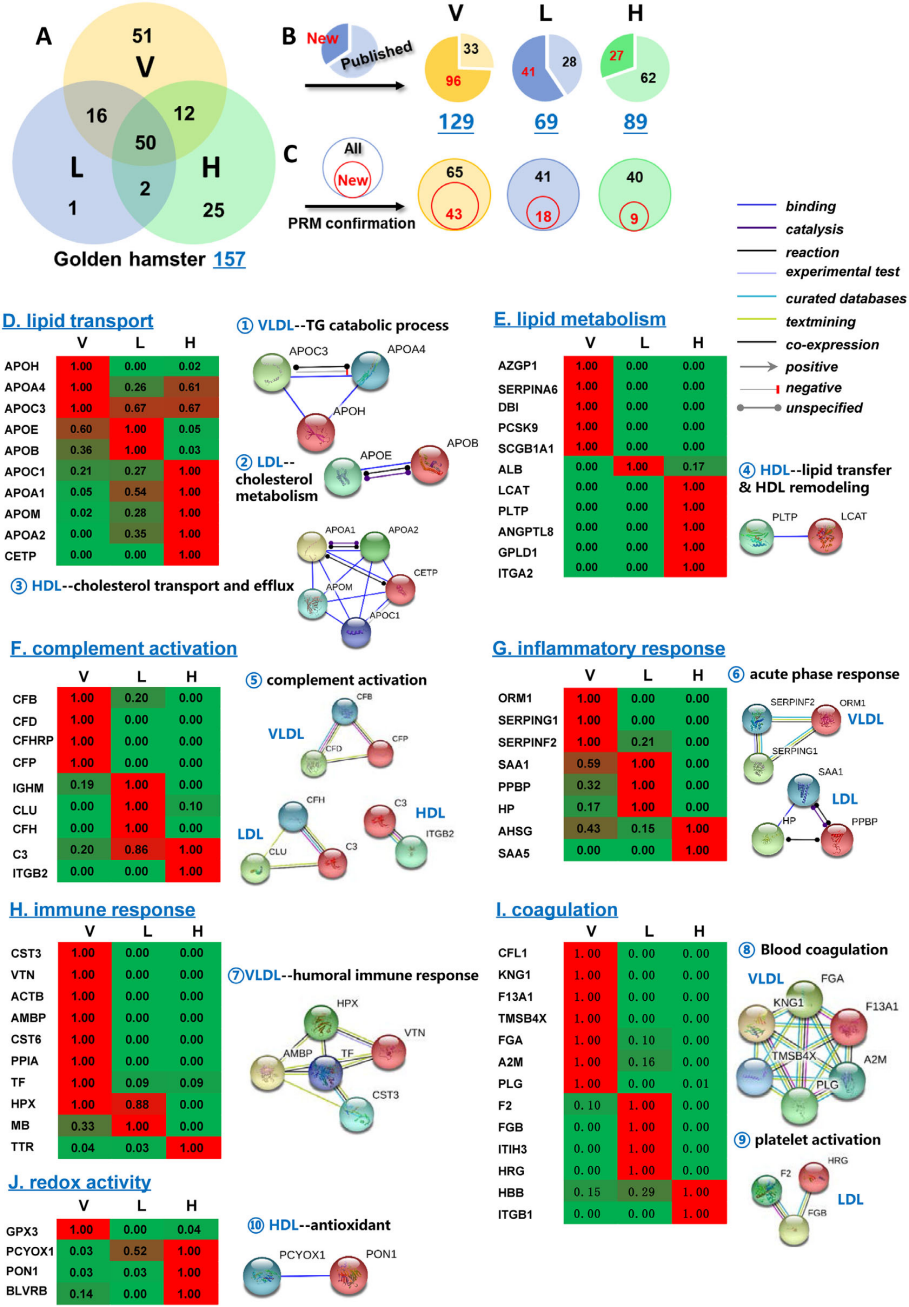
**Protein overlaps in VLDL/LDL/HDL of golden hamsters and their specific functions. (A)** Venn diagram of identified proteins in VLDL (V), LDL (L), and HDL (H) particles of golden hamsters. **(B)** Numbers of published (black) or newly detected (red) proteins compared with the VLDL/LDL/HDL proteome lists (Excel S1‐1/2/3). **(C)** Numbers of validated proteins by PRM proteomics. Black: total numbers; red: numbers of new proteins. **(D‐J)** Abundance heatmaps of 46 validated proteins in VLDL/LDL/HDL of normal golden hamsters using PRM targeted data. **Left**: Heatmaps of protein abundance. **Right**: Ten functional panels organized by biological functions and protein‐protein interaction in the STRING database. Each protein abundance in lipoprotein particles was from the normalized abundance by PRM quantitative proteomics. A value of 1.00 was assigned to the highest abundance of the specific protein among three lipoproteins, and other ratios were calculated accordingly. The highest value to the lowest are colored from red to green.

Functional analysis was undertaken using ingenuity pathway analysis (IPA) software, revealing that lipoprotein proteins focussed on four canonical pathways^6^ (LXR/RXR activation, FXR/RXR activation, acute phase response, clathrin‐mediated endocytosis signaling) and three core diseases (inflammatory response, organismal injury and abnormalities, cardiovascular disease) as reported,^6^ as shown in Figure S1.

Further analysis found that VLDL, LDL, and HDL had specific protein distribution with characteristic functions (Figure 1). Although lipoproteins are all responsible for lipid transport and metabolism (Figures 1D–1E), VLDL contained APOC3, APOA4, and APOH with the highest abundance performing TG catabolic processes. LDL contained apolipoprotein E (APOE) and apolipoprotein B‐100 (APOB) with the highest level mainly participated in cholesterol metabolism via binding to the LDL receptor. The APOA1, APOA2, APOC1, APOM, and CETP in HDL were responsible for cholesterol transport and efflux. Phosphatidylcholine‐sterol acyltransferase (LCAT) and phospholipid transfer protein (PLTP) in HDL played a core role in the lipid transfer and HDL remodeling (Figure 1E). The immunity group included complement activation, inflammatory response, and immune response (Figures 1F–1H). For complement activation, VLDL contained complement factor B (CFB), complement factor D (CFD), and properdin (CFP) with the highest abundance, while LDL contained the highest C3, CLU, and CFH. As for the acute‐phase response, VLDL mainly contained ORM1, SERPING1, and SERPINF2, while LDL included SAA1, HP, and PPBP. To perform blood coagulation, FGA, F13A1, A2M, PLG, TMSB4X, and KNG1 formed a function panel in VLDL (Figure 1I). Four proteins (GPX3 in VLDL, PCYOX1, PON1, and BLVRB in HDL) were related to redox activity (Figure 1J).

To investigate how the lipoprotein composition changes in hyperlipidemia, high‐fat diets were used for inducing the hyperlipidemic model. After 14 weeks, the plasma TC, LDL‐C, and lipid droplet accumulation significantly increased, supporting that hyperlipidemic model was established successfully (Table S3 and Figure S2).

Compared to the Con group, 55, 23, and 24 proteins were differentially expressed in the hyperlipidemic VLDL, LDL, and HDL particles, respectively (Figure S3). Notably, 30, 10, and 15 differential proteins of them were validated and showed the same change trend (Figure S5 and Excel S2). Functional analysis revealed that these differential proteins significantly regulate lipid metabolism, molecular transport, and small molecular biochemistry network (Figure 2). As shown in Figure 2B and Figure S4, hyperlipidemia mainly upregulated TG catabolic process in VLDL, especially APOC3 and APOA4, which are core regulators closely related to VLDL‐C and VLDL. In hyperlipidemic LDL, the cholesterol metabolism (APOB and APOE) were elevated and related to LDL‐C (Figure S4A). In HDL particles, hyperlipidemia significantly upregulated PLTP and LCAT, altering cholesterol transfer and HDL remodeling (Figure 2D). These results revealed that hyperlipidemia probably altered different function panels in lipoproteins, not single protein. Protein‐protein interaction networks were constructed using the STRING software (Figure S5A‐S5B), showing that proteins related to the innate immune response, stimulus response, and antioxidant functions were significantly downregulated, indicating that hyperlipidemia might affect immune response and redox activity. For the validated differential proteins, their change trends in diseases associated with hyperlipidemia were summarized in Table S5.

**FIGURE 2:**
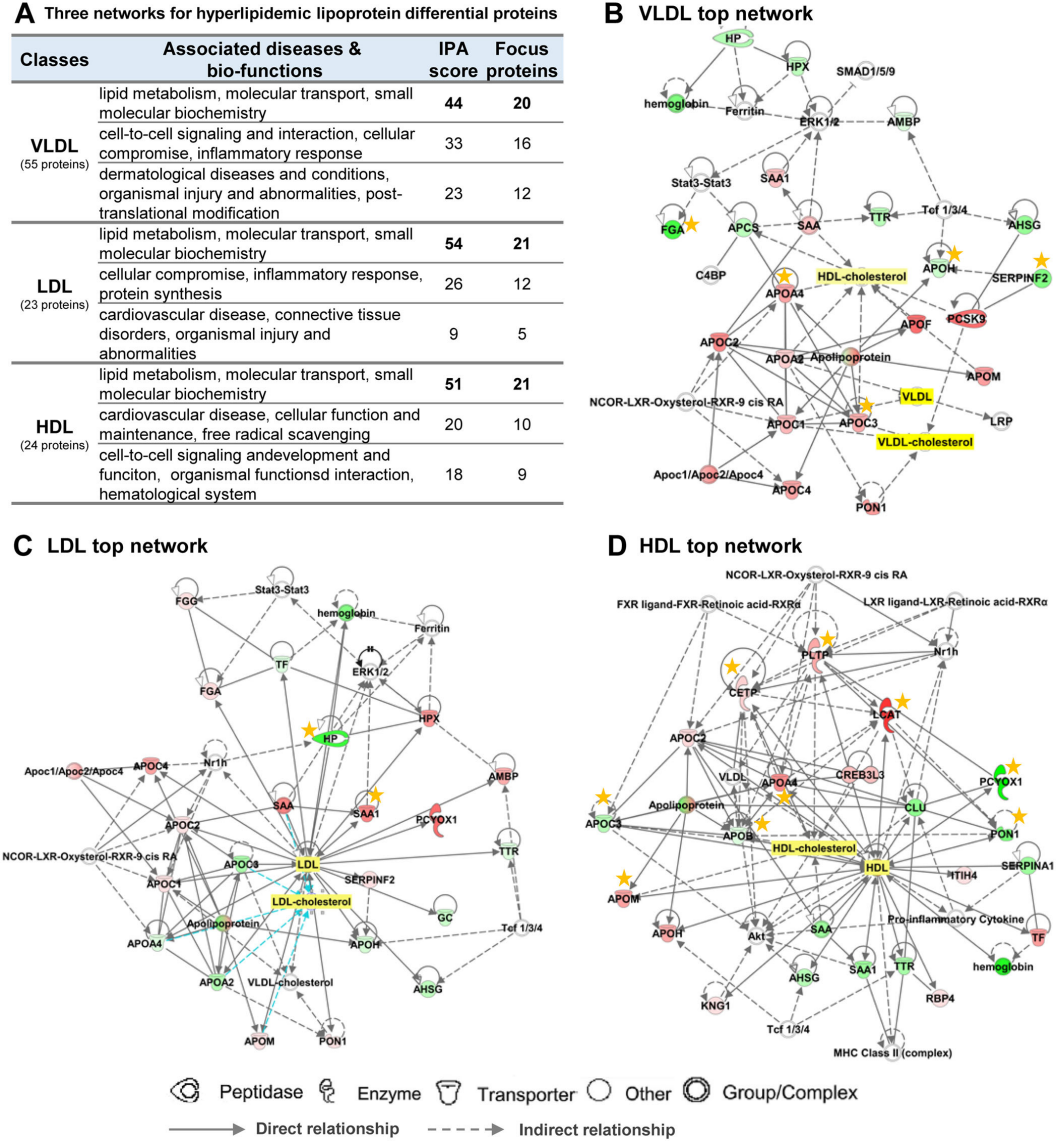
**The regulatory networks of VLDL, LDL, and HDL differential proteins in hyperlipidemic golden hamsters by IPA software with label‐free proteomic data. (A)** Top 3 networks associated with diseases and bio‐functions. **(B)** VLDL top network. **(C)** LDL top network. **(D)** VLDL top network. Red color: upregulated proteins; green color: downregulated proteins; orange star: validated differential proteins

Notably, 13 validated new proteins showed differential expression in hyperlipidemia including CFB, CFD, immune response proteins (SERPING1, ORM1, SERPINF2, CST3, TF), blood coagulation proteins (KNG1, FGA, A2M, CFL1) and antioxidant protein GPX3 (Figure S4). They are worthy of further investigation on function.

Importantly, the differential proteins in hyperlipidemia were also identified in human lipoproteins. We identified 119, 61, and 75 proteins in human VLDL, LDL, and HDL subclasses respectively, among which 60, 34, and 43 proteins shared in corresponding lipoproteins of golden hamsters and humans (Figure S6 and Table S4). Table 1 shows the similarity and specificity of lipoprotein proteome between humans and golden hamsters, containing components and functions. Additionally, we updated three human lipoprotein proteome databases compiled by the Davidson/Shah^9^ and Tomas Vaisar^10^ laboratory to January 2020 (Excel S1‐1/2/3), being the largest database.

**TABLE 1:** Biological functions of the identified proteins in VLDL/LDL/HDL from humans and golden hamsters

**Biological Functions**	**Both (73)**	**Human (61)**	**Golden Hamster (84)**
**1. Lipid metabolism**	**18**	**5**	**11**
1.1 Lipid transport	**(12): APOA2, APOA4, APOB, APOC1, APOA1, APOC3,** **APOE, APOH, APOM, CETP** , APOC2, APOC4	**(4)**: APOA5, APOD, APOF, APOL1	
1.2 Lipid metabolism	**(6): LCAT, PLTP, AZGP1, ALB,** LDLR, RBP4	**(1)**: LPA	**(11)**: DBI, BPIFA2, PSAP, ANGPTL8, SNCA, SERPINA6, PCSK9, SCGB1A1, POSTN, ITGA2, GPLD1
**2. Immunity**	**33**	**28**	**23**
2.1 Complement activation	**(5): C3, CFB, CFD, CLU, IGHM**	**(4)**: C4B, C9, CFHR4, C4BPA	**(9)**: C2, C4, CFH, CFI, CFP, ITGAM, ITGB2,CFP, CFHRP
2.2 Inflammatory response	**(14): AHSG, HP,ORM1, PPBP, SAA1**, SAA2, SERPINA1, SERPINC1, **SERPINF2, SERPING1**, THBS1, FN1, ITIH4, SERPINF1	**(6)**: AGT, IGFBP4, ORM2, PARK7, PF4, PLA2G7	(**5)**: APCS, ICAM1, VCAM1, SAA3, SAA5
2.3 Immune Response	**(14)**: A1BG, ACTB, **AMBP,** B2M, CAMP, CRP, **CST3,** GSN, **HPX**, KRT1, PPIA, **TF, TTR, VTN**	**(18)**: CD5L, CHGA, CNN2, COL1A1, IGHA1, IGHG1, IGHG2, IGHG3, IGHV3‐74, IGKC, IGLC2, JCHAIN, LBP, DEFA1, LRG1, NAPRT, UBB, SERPINA3	**(9)**: CRIP1, CST6, DSC1,IHH, KRT6A, MB, POMC, RPS27A, TREML1
**3. Coagulation**	**9**	**2**	**9**
	**(9): A2M, CFL1, F2, FGA, HBB, KNG1**, PLG, SERPIND1, F5	**(2)**: FBLN1, FLNA	**(9)**: F13A1, FGB, FGG, HRG, ITGB1, ITIH3, KLKB1, TFPI, TMSB4X
**4. Redox activity**	**3**		**4**
	**(3): PCYOX1, PON1,GPX3**		**(4)**: PRDX2, BLVRB, CP, TRX
**5. Others**	**10**	**26**	**38**
	**(10)**: ANG, ARHGDIB, **HBA**, ITIH2, **KRT2, PFN1, SFTPB,** TXN, **VTDB,** KRT10	**(26)**: AHNAK, BASP1, CCDC40, CSRP1, FERMT3, HPR, IGFBP2, IGFBP3, KRT33B, KRT83, KRT9, MENT, MTPN, OTOF, PDLIM1, PON3, POTEI, RNASE4, SAA2‐SAA4, , and so on.	**(37)**: AFM, ANTXR1, ARHGDIB, CA1, TNA, CNDP1, CST6, EIF5A, FAM171B, FETUB, GC, HBA, HBZ, HPP, ITIH1, ITIH4, and so on.

For 73 shared proteins, proteins marked in red and bold were new in lipoproteins and validated by PRM analysis, while the proteins marked in black and bold are both reported and validated. The detailed description is in **Table S1/S4**

Our study provides valuable lipoprotein proteome resources for hyperlipidemia. Global profiling and functional analysis of lipoproteins revealed that hyperlipidemia altered 30, 10, and 15 abnormal proteins of VLDL, LDL, and HDL, including 13 new proteins, giving us new insights that hyperlipidemia affect immunity and redox activity. It suggests that the relationship of immunity or antioxidant activity to hyperlipidemia or cardiovascular disease and their therapies need further investigation.

## CONFLICT OF INTEREST

The authors declare no competing financial interest.

## AVAILABILITY OF DATA AND MATERIALS

All materials and data are available.

## AUTHOR CONTRIBUTIONS

All authors contributed to our research. Jinlan Zhang, Miao Lin and Menglin Li designed the study and finished the manuscript. Funding acquisition was by Jinlan Zhang. Literature search, experiments, and data collection and analysis were finished by Miao Lin, Menglin Li, Hao Zheng, and Haidan Sun.

## Supporting information

SUPPORTING INFORMATION

SUPPORTING INFORMATION

SUPPORTING INFORMATION

SUPPORTING INFORMATION

SUPPORTING INFORMATION

SUPPORTING INFORMATION

SUPPORTING INFORMATION
