# Supplementary material for: Lipoprotein proteome profile: novel insight into hyperlipidemia
Source: Clin Transl Med. 2021 Apr 8;11(4):e361. doi: 10.1002/ctm2.361 (PMC8032137; doi:10.1002/ctm2.361)
Supplement: Supplementary file 1 — SUPPORTING INFORMATION [file CTM2-11-e361-s005.docx]

**Supplementary material**

**Lipoprotein proteome profile: novel insight into hyperlipidemia**

**Miao Lin ^# 1^, Meng-Lin Li ^# 1^, Hao Zheng ^1^, Haidan Sun ^2^, Jin-Lan Zhang * ^1^**

**Institute:** 1. State Key Laboratory of Bioactive Substances and Functions of Natural Medicines, Institute of Materia Medica, Chinese Academy of Medical Sciences *&* Peking Union Medical College, Beijing 100050, P. R. China.

2. Core facility of instrument, Institute of Basic Medical Sciences, Chinese Academy of Medical Sciences, School of Basic Medicine, Peking Union Medical College, Beijing 100005, China.

^#^ These authors have contributed equally to this work.

**^∗^ Corresponding author:** Prof. Jin-Lan Zhang, Tel.: +86-10-83154880; Fax: +86-10-63017757

E-mail: [zhjl@imm.ac.cn](https://mail.imm.ac.cn/owa/redir.aspx?C=13XQR-VDkTbLxaSZWyqaLyeHyVjeZjDbd7KC5vcSqlmRSKgLtpDUCA..&URL=mailto%3azhjl%40imm.ac.cn)

State Key Laboratory of Bioactive Substances and Functions of Natural Medicines, Institute of Materia Medica, Chinese Academy of Medical Sciences and Peking Union Medical College, 2 Nanwei Road, Beijing 100050, China.

# Methods and materials

***Materials and Reagents***

Trypsin and rLys-C (MS grade) were obtained from Promega Corporation (Madison, WI). High-fat diets (HFD; D12492, 60 kcal% fat) were from Research Diets (New Brunswick, NJ). Total cholesterol (TC), HDL-cholesterol (HDL-C), triglyceride (TG), and LDL-cholesterol (LDL-C) enzymatic reagents were purchased from BioSino Bio-Technology and Science Inc (Beijing, China). LC/MS-grade methanol (MeOH), acetonitrile (ACN), isopropanol (IPA), and water were obtained from Thermo Fisher Scientific (Waltham, MA). Unless otherwise specified, all other reagents were obtained from Sigma Aldrich (St. Louis, MO) at HPLC grade.

***Animal Experiments, Biochemical Analysis and Histopathology***

Male Golden Syrian hamsters (*Mesocricetus auratus*; 8 weeks; n = 22), purchased from Vital River Laboratory Animal Technology Co. Ltd. (Beijing, China), were randomly divided into control (Con) and model (Mod) groups (n = 11). All the procedures involving animals were approved by the Laboratories Institutional Animal Care and Use Committee of the Chinese Academy of Medical Sciences and Peking Union Medical College, and all the experimental protocols were performed using the approved guidelines.

The Con group was fed with normal chow, and the Mod group was fed with HFD. After 14 weeks (1 week for ), plasma and liver tissues were collected with removing the feed in cheek pouches and 12 h fasting. And 100 μl plasma was used to measure TC, TG, LDL-C, and HDL-C, while the remainder was pooled at equal volumes and then was used for lipoprotein separation. Portions of liver tissue were used for hematoxylin and eosin (H&E) and oil red O staining at the Biological Analysis Center of Institute of Materia Medical & Chinese Academy of Medical Science. All the biological samples were stored at -80℃.

***Lipoprotein Isolation and Preparation***

Human serum were obtained from 3 healthy volunteers aged 22 to 30 years. To alleviate individual variation, 3 human serum samples, 11 plasma samples from the Con or Mod groups were pooled together to isolate the three lipoproteins for label-free proteomic analysis. For PRM targeted validation, 3 individual human serum samples and 4 individual plasma samples from the Con and Mod groups were applied, respectively.

VLDL, LDL, and HDL were separated by using an established sequential density gradient ultracentrifugation (UC) method described by Lebrilla *et al*. with minor modifications ^1,2^. Fresh potassium bromide (KBr) solutions with the densities (d) of 1.019, 1.063, 1.210, and 1.340 g/ml were prepared and verified using a BHDM-YM08 Benchtop densitometer (readability, 0.0001 g/ml; Baiheng, China). Briefly, pooled or individual plasma samples (1.9 ml) were adjusted to d = 1.019 by adding concentrated KBr solution (d = 1.340), and then added KBr solution (d = 1.019) for a final volume of 4.7 ml tube capacity (OptiSeal, Beckman Coulter). Ultracentrifugation was performed using a Beckman Optima XPN100 system equipped with a TLA-110 fixed-angle rotor (Beckman Coulter) for 2.25 h, at 600,000 g and 15 °C. The VLDL fraction (1.9 ml; d < 1.019) was collected from the top of the tube. The remainder was adjusted to d = 1.063 and overlain by KBr solution (d = 1.063) followed by ultracentrifugation for 3.2 h. The supernatant LDL fraction (1.9 ml; 1.019 < d < 1.063) was collected. The remainder was adjusted to d = 1.210. Next, the sample was divided into two equal volumes, overlain by KBr solution (d = 1.210), and ultracentrifuged for 3.3 h. The HDL fractions (top 1.0 ml; 1.063 < d < 1.210) were collected. All densities were measured by the densitometer.

All lipoprotein fractions were subjected to KBr removal using the Amicon ultra-3K centrifugal filter device (Merck Millipore, MA), desalted with phosphate-buffered saline (PBS, pH 7.45), and concentrated to 50 μl. Then the samples was added 1 ml CHCl_3_-MeOH (2:1, *v/v*), vortexed for 5 min, and centrifuged at 13,000 rpm for 10 min at 10 °C. The protein sediments were collected to proteomic analysis.

***Proteomic Analysis***

**Digestion.** The BCA protein assay kit (Thermo Scientific) was to determine the total protein concentration. Aliquots of VLDL, LDL, and HDL (100 μg of protein) were digested according to the filter-aided sample preparation method ^3^ under optimized reduction and alkylation conditions. Aliquots of VLDL, LDL, and HDL (100 μg of protein) were digested according to the filter-aided sample preparation (FASP) method under optimized reduction and alkylation conditions. VLDL and LDL proteins were reduced with 75 mM dithiothreitol (DTT) at 56 °C in 50 mM NH_4_HCO_3_ for 1 h and alkylated with 150 mM iodoacetamide (IAA) in 50 mM NH_4_HCO_3_ for 30 min in the dark at room temperature. HDL proteins were incubated in 25 mM DTT at 56 °C for 1 h and alkylated in 50 mM IAA in the dark for 30 min. Each sample was digested with Lys-C (100:1, w/w) in 6 M urea buffer for 4 h at 37 °C and was diluted in 1 M urea for trypsin (50:1, w/w) digestion overnight. The reaction was quenching with 5 μl of formic acid. The peptides were desalted using solid-phase extraction (SPE) columns (Oasis PRIME HLB; Waters, Rydalmere, NSW, Australia) and drying with nitrogen.

**Label-free proteomic analysis.** After drying, the peptides were dissolved with 0.1% FA and 2% ACN in H_2_O (0.25 μg/μl) and were subjected to reversed-phase nanoLC-MS/MS analysis using the EASY-nLC 1000 system coupled to an Orbitrap Fusion Lumos Tribrid mass spectrometer (Thermo Scientific, Bremen, Germany). Individual samples containing 1 μg of peptide mixture were preconcentrated on a trap column (Acclaim PepMap 100; C18; 3 μm; 100 Å; 75 μm × 2 cm) and then were separated on an analytical column(Acclaim PepMap RSLC column; C18; 2 μm; 100 Å; 75 μm × 25 cm) with a gradient of 60 min (solvent A: 0.1% FA, 2% ACN in water; solvent B: 0.1% FA in ACN; 0–3 min, 4% B; 3–44 min, 4%–20% B; 44–53 min, 20%–28% B; 53–58 min, 28%–95% B; 58–60 min, 95% B; flow rate, 300 nL/min). The data were acquired using data-dependent acquisition (DDA). Full-scan MS spectra were acquired using the Orbitrap system at 60,000 resolution (*m/z* 350–1500) and an automatic gain control (AGC) target value of 1×10^5^ charges with a maximum injection time of 50 ms. High-energy collisional dissociation (HCD) fragmentation was recorded at 15,000 resolution using a normalized collision energy of 32% for all scans and an AGC target value of 2×10^4^ charges with a max injection time of 30 ms.

**PRM targeted proteomics analysis.** To validate the proteins newly assigned to lipoproteins and the differential proteins, PRM analysis was performed using the TripleTOF 5600 system (SCIEX, Framingham, MA, USA) interfaced with the ACQUITY UPLC system (Waters, MA, USA). Owing to inadequate plasma samples, the lipoprotein fractions were isolated from 4 golden hamsters of each group and were digested as described in the above methods. The peptides were separated using an Acclaim PepMap RSLC column (C18; 2 μm; 100 Å; 75 μm × 25 cm) with an eluted gradient for 60 min (solvent A: 0.1% FA, 2% ACN in water; solvent B: 0.1% FA in ACN; 0–40 min, 5%–20% B; 40–44 min, 20%–30% B; 44–45 min, 30%–95% B; 45–50 min, 95% B; 50–50.1 min, 95%–5% B; 50.1–60 min, 5% B; flow rate, 600 nL/min). The MS^1^ spectra were obtained from 350 to 1500 *m/z* with charge states of +2 and +3, while MS/MS spectra were acquired from 100 to 1250 *m/z* in the high-resolution mode.

**Data analysis.** All proteomic data were deposited into iProX resources center ^4^ with the dataset identifier IPX0001366000. To identify the proteins, the MS/MS spectra of label-free analysis were converted from RAW files to the Mascot generic files (.mgf) via Proteome Discoverer 2.1 (Thermo Scientific, Waltham, USA). The mgf files were searched using Mascot software (V.2.6.0Matrix Science, London, UK) against the Uniport Proteome database restricted to *Mesocricetus auratus* (released at 2017.04.13; 31,693 protein entries). The search parameters were set as follows: the precursor mass accuracy was set to 8 ppm, the MS/MS accuracy was set to 0.03 Da, carbamidomethylation (C) was set as a fixed modification, oxidation (M) and carbamyl (Lys) were set as variable modifications, trypsin was specified as the digestion enzyme, and a maximum of two missed cleavages was allowed. Scaffold Q^+^ software (version: 4.6.1, Proteome Software Inc., Portland, OR) was used to integrate and validate the peptide and protein identifications based on the standard legacy Peptide Prophet scoring system 20. Following the Peptide Prophet algorithm ^5^, peptide identifications were accepted if they established at greater than 99.0% probability. Additionally, a protein was positively identified only when it achieved the peptides and proteins false discovery rate (FDR) was less than 1% and was identified at least two unique peptides.

Progenesis QI for proteomics software (V2.4, Newcastle upon Tyne, UK) was used for label-free quantification based on ion-intensity ^6^. The profile data of the MS scans and MS/MS scans were transformed into peak lists with Progenesis QI using a personal peak-modeling algorithm. The default parameters of the peak selection and alignment algorithm were used. Statistical analysis of label-free quantification was performed by unpaired Student's t-test implemented in Progenesis QI software to select differentially expressed proteins, while *p*-values were adjusted using the Benjamini-Hochberg correction. Proteins with a fold change ≥ 2-fold and *p*-value ≤ 0.05 were considered as differential expression between two groups. Heatmap analysis and hierarchical clustering of differential proteins were constructed by TBtools ^7^. Gene Ontology (GO) annotation of proteins was performed using UniProt Knowledgebase (UniProtKB). The network of protein interactions was performed on the STRING (<http://string-db.org>) software with setting an interaction score > 0.900. The SwissProt accession numbers were inputted into the ingenuity pathway analysis (IPA) software to perform disease/bio-functions and regulatory networks.

Skyline software (V4.2) was applied to the PRM analysis and calculate the peptide abundance for each protein ^8^. The Biognosys iRT standard peptides (as IS, Schlieren, Switzerland) were added to normalize the retention time and protein abundance. For PRM analysis, the raw data of label-free analysis were first processed using Mascot software and searched against the *Mesocricetus auratus* proteome database (UP000189706). All results and raw data were imported to Skyline software (V4.2) to generate a protein library. VLDL, LDL, and HDL generated independent libraries. Next, 1-3 unique peptides and corresponding transitions of targeted proteins were selected. It depending on the following criteria: maximum missed cleavages ≤ 2; peptide length between 8 and 25; enzyme set as trypsin [KR/P]; detection frequency > 50%; cysteine carbamidomethylation as the variable modification; precursor charge setting as 2 and 3; ion charges setting as 1 and 2; and ion types setting as b, y. The Biognosys iRT standard peptides (as IS, Schlieren, Switzerland) were added to normalize the retention time and protein abundance. Skyline software was used to calculate the peptide abundance for each protein. The normalized protein abundance was presented as the sum of the transition intensities of a peptide after IS peptide abundance normalization.

| **Table S1. Proteins identified in three lipoproteins of golden hamster plasma by label-free proteomics and validated by PRM targeted proteomics** | | | | | | | | | |
| --- | --- | --- | --- | --- | --- | --- | --- | --- | --- |
| **No.** | **Protein name (157)** | **Accession*** | **GeneID** | **VLDL** | | **LDL** | | **HDL** | |
|  |  |  |  | **Our study** | **33 R ( 20 R^a^), 96 N (43 ^PRM^)** | **Our study** | **28 R ( 17 R^a^), 41 N (18 ^PRM^)** | **Our study** | **62 R ( 55 R^a^), 27 N (9 ^PRM^)** |
| 1 | actin, cytoplasmic 1 | A0A1U7Q273_MESAU | Actb | 1^PRM^ | R^a^ | 1 | N | 1 | N |
| 2 | acyl-CoA-binding protein | A0A1U7QBI0_MESAU | Dbi | 1^PRM^ | N | -- | -- | -- | -- |
| 3 | afamin | A0A1U8BMK2_MESAU | Afm | 1 | N | -- | -- | -- | -- |
| 4 | alpha-1-acid glycoprotein 1 | A0A1U7QUU5_MESAU | Orm1 | 1^PRM^ | N | -- | -- | -- | -- |
| 5 | alpha-1-antitrypsin | A0A1U8BK25_MESAU | Serpina1 | 1 | R^a^ | 1 | R^a^ | 1 | R^a^ |
| 6 | alpha-1B-glycoprotein | A0A1U8C678_MESAU | A1bg | 1 | R | -- | -- | -- | -- |
| 7 | alpha-1-inhibitor 3 | A0A1U8BEB6_MESAU | LOC101843823, A1i3 | 1 | N | 1 | N | -- | -- |
| 8 | alpha-2-antiplasmin | A0A1U8BM09_MESAU | Serpinf2 | 1^PRM^ | N | 1^PRM^ | N | 1 | R^a^ |
| 9 | alpha-2-HS-glycoprotein | A0A1U8BVC5_MESAU | Ahsg | 1^PRM^ | R | 1^PRM^ | R | 1^PRM^ | R^a^ |
| 10 | alpha-2-macroglobulin | A0A1U8CUG0_MESAU | A2m | 1^PRM^ | N | 1^PRM^ | N | 1 | R^a^ |
| 11 | alpha-synuclein | A0A1U8C123_MESAU | Snca | 1 | N | 1 | N | -- | -- |
| 12 | aminopeptidase N | A0A1U7R1Z8_MESAU | Anpep | -- | -- | 1 | N | 1^PRM^ | R |
| 13 | angiogenin | A0A1U8CQE2_MESAU | Ang | 1^PRM^ | N | -- | -- | -- | -- |
| 14 | angiopoietin-like protein 8 | A0A1U7QT44_MESAU | Angptl8 | -- | -- | -- | -- | 1^PRM^ | Ra |
| 15 | anthrax toxin receptor 1 | A0A1U7QCT3_MESAU | Antxr1 | -- | -- | -- | -- | 1^PRM^ | R |
| 16 | anthrax toxin receptor 2 | A0A1U7QAQ9 _MESAU | Antxr2 | -- | -- | 1 | N | 1 | R |
| 17 | antithrombin-III | A0A1U8BR94_MESAU | Serpinc | 1 | N | 1 | N | 1 | R^a^ |
| 18 | apolipoprotein A-I | A0A1U7Q2B6_MESAU | Apoa1 | 1^PRM^ | R^a^ | 1^PRM^ | R^a^ | 1^PRM^ | R^a^ |
| 19 | apolipoprotein A-II | A0A1U7R7S5_MESAU | Apoa2 | 1^PRM^ | N | 1^PRM^ | R^a^ | 1^PRM^ | R^a^ |
| 20 | apolipoprotein A-IV | A0A1U7QMM4_MESAU | Apoa4 | 1^PRM^ | R^a^ | 1^PRM^ | R^a^ | 1^PRM^ | R^a^ |
| 21 | apolipoprotein B-100 | A0A1U7QU07_MESAU | Apob | 1^PRM^ | R^a^ | 1^PRM^ | R^a^ | 1^PRM^ | R^a^ |
| 22 | apolipoprotein C-I | A0A1U7QUM6_MESAU | Apoc1 | 1^PRM^ | R^a^ | 1^PRM^ | R^a^ | 1^PRM^ | R^a^ |
| 23 | apolipoprotein C-II | A0A1U8CMV9_MESAU | Apoc2 | 1 | R^a^ | 1 | R^a^ | 1 | R^a^ |
| 24 | apolipoprotein C-III | A0A1U7QK70_MESAU | Apoc3 | 1^PRM^ | R^a^ | 1^PRM^ | R^a^ | 1^PRM^ | R^a^ |
| 25 | apolipoprotein C-IV | A0A1U7R5I0_MESAU | Apoc4 | 1 | R^a^ | -- | -- | -- | -- |
| 26 | apolipoprotein E | A0A1U7QUL7_MESAU | Apoe | 1^PRM^ | R^a^ | 1^PRM^ | R^a^ | 1^PRM^ | R^a^ |
| 27 | apolipoprotein M | A0A1U7QVF5_MESAU | Apom | 1^PRM^ | R^a^ | 1^PRM^ | R^a^ | 1^PRM^ | R^a^ |
| 28 | beta-2-glycoprotein 1 | A0A1U7QB41_MESAU | Apoh | 1^PRM^ | R | 1^PRM^ | R | 1^PRM^ | R^a^ |
| 29 | beta-2-microglobulin | A0A1U7QLF7_MESAU | B2m | 1 | N | -- | -- | -- | -- |
| 30 | beta-Ala-His dipeptidase | A0A1U8CLW4_MESAU | Cndp1 | 1 | N | -- | -- | 1^PRM^ | N |
| 31 | BPI fold-containing family A member 2 | Q6YBQ8_MESAU | Bpifa2 | -- | -- | -- | -- | 1 | N |
| 32 | BPI fold-containing family B member 3 | A0A1U8CZ95_MESAU | LOC101823870 | -- | -- | -- | -- | 1^PRM^ | N |
| 33 | carbonic anhydrase 1 | A0A1U7QIM1_MESAU | Ca1 | 1^PRM^ | N | -- | -- | 1 | N |
| 34 | cathelicidin antimicrobial peptide | A0A1U7QJA2_MESAU | Camp | 1 | R^a^ | -- | -- | 1 | R^a^ |
| 35 | C-C motif chemokine 6 | A0A1U7QQI8_MESAU | LOC101843608, Ccl20 | 1^PRM^ | N | 1^PRM^ | N | 1 | N |
| 36 | ceruloplasmin | A0A1U7R5I7_MESAU | Cp | 1 | N | -- | -- | -- | -- |
| 37 | cholesteryl ester transfer protein | A0A1U7QGU4_MESAU | Cetp | -- | -- | -- | -- | 1^PRM^ | R^a^ |
| 38 | clusterin | A0A1U8C2W4_MESAU | Clu | 1 | R^a^ | 1^PRM^ | R^a^ | 1^PRM^ | R^a^ |
| 39 | coagulation factor V | A0A1U7Q728_MESAU | F5 | 1 | N | -- | -- | -- | -- |
| 40 | coagulation factor XIII A | A0A1U7Q8V5_MESAU | F13a1 | 1^PRM^ | N | -- | -- | -- | -- |
| 41 | cofilin-1 | A0A1U7QYH2_MESAU | Cfl1 | 1^PRM^ | N | -- | -- | -- | -- |
| 42 | complement C2 | A0A1U7R3Q2_MESAU | C2 | 1 | N | -- | -- | -- | -- |
| 43 | complement C3 | A0A1U8CME2_MESAU | C3 | 1^PRM^ | R^a^ | 1^PRM^ | R^a^ | 1^PRM^ | R^a^ |
| 44 | complement C4 | A0A1U7RGL4_MESAU | LOC101830930, C4 | 1 | N | 1 | N | 1 | N |
| 45 | complement factor B | A0A1U7RGK9_MESAU | Cfb | 1^PRM^ | N | 1^PRM^ | N | 1 | R^a^ |
| 46 | complement factor D | A0A1U8CRJ7_MESAU | Cfd | 1^PRM^ | N | -- | -- | 1 | R^a^ |
| 47 | complement factor H | A0A1U8BYV0_MESAU | Cfh | 1 | N | 1^PRM^ | N | -- | -- |
| 48 | complement factor H-related protein 1 | A0A1U8BVN6_MESAU | Cfhrp | 1^PRM^ | N | 1^PRM^ | N | 1 | N |
| 49 | complement factor I | A0A1U8CF62_MESAU | Cfi | 1 | N | -- | -- | -- | -- |
| 50 | corticosteroid-binding globulin | A0A1U7Q9B1_MESAU | Serpina6, Cbg | 1^PRM^ | N | -- | -- | 1 | N |
| 51 | C-reactive protein | A0A1U7QPL0 _MESAU | Crp | -- | -- | -- | -- | 1 | R |
| 52 | cystatin-C | A0A1U7Q8B2_MESAU | CST3 | 1^PRM^ | N | -- | -- | -- | -- |
| 53 | cystatin-M | A0A1U7Q2V1_MESAU | Cst6 | 1^PRM^ | N | -- | -- | -- | -- |
| 54 | cysteine-rich protein 1 | A0A1U7QIK1_MESAU | LOC101822555, Crip1 | 1 | N | -- | -- | -- | -- |
| 55 | cytochrome b5 | A0A1U7QSD8_MESAU | LOC101833334 | 1 | N | -- | -- | -- | -- |
| 56 | desmocollin-1 | A0A1U7Q3D0_MESAU | Dsc1 | 1 | N | -- | -- | -- | -- |
| 57 | eukaryotic translation initiation factor 5A-1 | A0A1U7QHC7_MESAU | LOC101840102 | 1 | N | -- | -- | -- | -- |
| 58 | extracellular matrix protein 1 | A0A1U7R2H7_MESAU | Eif5a | 1 | N | -- | -- | -- | -- |
| 59 | fetuin-B | A0A1U7QP51_MESAU | Fetub | 1^PRM^ | N | 1 | N | 1^PRM^ | N |
| 60 | fibrinogen alpha chain | A0A1U7QP17_MESAU | Fga | 1^PRM^ | R^a^ | 1^PRM^ | R^a^ | 1 | R^a^ |
| 61 | fibrinogen beta chain | A0A1U7QCL5_MESAU | Fgb | 1 | N | 1^PRM^ | N | 1 | R^a^ |
| 62 | fibrinogen gamma chain isoform X1 | A0A1U7QWI4_MESAU | Fgg | 1 | R^a^ | 1 | R^a^ | -- | -- |
| 63 | fibronectin | A0A1U7Q673_MESAU | Fn1 | 1 | N | 1 | N | 1 | R^a^ |
| 64 | flavin reductase | A0A1U7RG83_MESAU | Blvrb | 1^PRM^ | N | -- | -- | 1^PRM^ | N |
| 65 | gelsolin | A0A1U7R6U4_MESAU | Gsn | 1 | N | 1 | N | 1 | R^a^ |
| 66 | glutathione peroxidase 3 | A0A1U8BIS9_MESAU | Gpx3 | 1^PRM^ | N | -- | -- | 1^PRM^ | N |
| 67 | guanylate-binding protein 6 | A0A1U8C1S7_MESAU | LOC101823391 | -- | -- | -- | -- | 1 | N |
| 68 | haptoglobin | A0A1U7Q238_MESAU | Hp | 1^PRM^ | N | 1^PRM^ | R | -- | -- |
| 69 | Hemoglobin subunit alpha | P01945_MESAU | Hba | 1^PRM^ | R | 1^PRM^ | R | 1^PRM^ | R^a^ |
| 70 | Hemoglobin subunit beta | P02094_MESAU | Hbb | 1^PRM^ | R | 1^PRM^ | N | 1^PRM^ | R^a^ |
| 71 | Hemoglobin subunit beta | A0A3Q0CX13_MESAU | LOC101833678 | -- | -- | -- | -- | 1^PRM^ | R |
| 72 | hemoglobin subunit zeta | A0A1U7R5T9_MESAU | Hbz | 1^PRM^ | N | -- | -- | 1 | N |
| 73 | hemopexin | A0A1U7RF88_MESAU | Hpx | 1^PRM^ | N | 1^PRM^ | N | -- | -- |
| 74 | heparin cofactor 2 | A0A1U7QNI5_MESAU | Serpind1 | 1 | N | -- | -- | -- | -- |
| 75 | histidine-rich glycoprotein | A0A1U7QP95_MESAU | Hrg | 1 | R | 1^PRM^ | N | -- | -- |
| 76 | Ig mu chain C region | P06337_MESAU | Ighm | 1^PRM^ | R | 1^PRM^ | N | -- | -- |
| 77 | indian hedgehog protein | A0A1U8BPJ9_MESAU | Ihh | -- | -- | -- | -- | 1 | N |
| 78 | inhibitor of carbonic anhydrase | A0A1U8BW24_MESAU | LOC101828453 | 1 | N | -- | -- | -- | -- |
| 79 | inositol monophosphatase 3 | A0A1U7QFY8_MESAU | Impad1 | -- | -- | -- | -- | 1 | R^a^ |
| 80 | insulin-like growth factor-binding protein complex acid labile subunit | A0A1U7QLS9_MESAU | Igfals | -- | -- | -- | -- | 1^PRM^ | N |
| 81 | integrin alpha-2 | A0A1U7RA21_MESAU | Itga2 | -- | -- | -- | -- | 1^PRM^ | R |
| 82 | integrin alpha-M | A0A1U7Q562 _MESAU | Itgam | -- | -- | -- | -- | 1 | N |
| 83 | integrin beta-1 | A0A1U8CGG0_MESAU | Itgb1 | -- | -- | -- | -- | 1^PRM^ | R^a^ |
| 84 | integrin beta-2 | A0A1U8BT74_MESAU | Itgb2 | -- | -- | -- | -- | 1^PRM^ | N |
| 85 | Inter-alpha-trypsin inhibitor heavy chain H1 | P97278_MESAU | Itih1 | 1 | N | -- | -- | -- | -- |
| 86 | inter-alpha-trypsin inhibitor heavy chain H2 | A0A1U7Q2C1_MESAU | Itih2 | 1 | N | 1 | N | -- | -- |
| 87 | inter-alpha-trypsin inhibitor heavy chain H3 | A0A1U7Q227_MESAU | Itih3 | 1 | N | 1^PRM^ | N | -- | -- |
| 88 | inter-alpha-trypsin inhibitor heavy chain H4 isoform X3 | A0A1U8CKM1_MESAU | Itih4 | 1 | R | 1 | R | 1 | R^a^ |
| 89 | intercellular adhesion molecule 1 | A0A1U7QQC7_MESAU | Icam1 | -- | -- | -- | -- | 1 | N |
| 90 | keratin, type I cuticular Ha4 | A0A3Q0CFY7_MESAU | Krt34 | 1 | N^b^ | -- | -- | -- | -- |
| 91 | keratin, type I cuticular Ha5 | A0A1U7QWJ1_MESAU | Krt35 | 1 | N^b^ | -- | -- | -- | -- |
| 92 | keratin, type I cytoskeletal 10 | A0A1U8C3P8_MESAU | Krt10 | 1 | N | 1 | N | 1 | R^a^ |
| 93 | keratin, type I cytoskeletal 14 | A0A1U7R4L9_MESAU | Krt14 | 1 | N^b^ | 1 | N^b^ | 1 | N^b^ |
| 94 | keratin, type I cytoskeletal 18 | A0A1U7QJF8_MESAU | Krt18 | 1 | N^b^ | -- | -- | -- | -- |
| 95 | keratin, type II cuticular Hb1 | A0A1U7QGY6_MESAU | LOC101832771 | 1 | N^b^ | -- | -- | -- | -- |
| 96 | keratin, type II cytoskeletal 1 | A0A1U7QJF3_MESAU | Krt1 | 1 | N^b^ | 1 | N | 1 | R^a^ |
| 97 | keratin, type II cytoskeletal 2 | A0A1U7Q9U1_MESAU | Krt2 | 1 | N^b^ | 1^PRM^ | N | -- | -- |
| 98 | keratin, type II cytoskeletal 5 | A0A1U7Q422_MESAU | Krt5 | 1 | N^b^ | 1 | N^b^ | -- | -- |
| 99 | keratin, type II cytoskeletal 6A | A0A3Q0CFL1_MESAU | Krt6a | 1 | N | 1 | N | 1 | R^a^ |
| 100 | kininogen-1 | A0A1U8BRY4_MESAU | Kng1 | 1^PRM^ | N | 1 | N | 1 | R^a^ |
| 101 | low-density lipoprotein receptor | A0A1U7QT49_MESAU | Ldlr | 1 | R | -- | -- | -- | -- |
| 102 | lumican | A0A1U7Q4A8_MESAU | Lum | 1^PRM^ | N | -- | -- | -- | -- |
| 103 | lysozyme C-1 | A0A1U8CNI4_MESAU | Lyz1 | 1^PRM^ | N | -- | -- | -- | -- |
| 104 | mannose-binding protein A | A0A1U7R7C1_MESAU | LOC101835615 | 1 | N | -- | -- | -- | -- |
| 105 | myoglobin | A0A1U7QU54_MESAU | Mb | 1^PRM^ | N | 1^PRM^ | N | 1 | N |
| 106 | parvalbumin alpha | A0A1U8BK18_MESAU | Pvalb | 1^PRM^ | N | -- | -- | -- | -- |
| 107 | peptidyl-prolyl cis-trans isomerase A | A0A1U7RE28_MESAU | Ppia | 1^PRM^ | N | -- | -- | -- | -- |
| 108 | periostin | A0A1U7QYR1_MESAU | Postn | 1 | N | -- | -- | -- | -- |
| 109 | peroxiredoxin-2 | A0A1U7RHE5_MESAU | Prdx2 | 1 | N | -- | -- | -- | -- |
| 110 | phosphatidylcholine-sterol acyltransferase | A0A1U7QCW4_MESAU | Lcat | -- | -- | -- | -- | 1^PRM^ | R^a^ |
| 111 | phosphatidylinositol-glycan-specific phospholipase D | A0A1U8BF27_MESAU | Gpld1 | -- | -- | -- | -- | 1^PRM^ | R^a^ |
| 112 | phospholipid transfer protein | A0A1U7RB45_MESAU | Pltp | -- | -- | -- | -- | 1^PRM^ | R^a^ |
| 113 | pigment epithelium-derived factor | A0A1U7Q9Y5_MESAU | Serpinf1 | -- | -- | -- | -- | 1 | R^a^ |
| 114 | kallikrein | A0A1U7Q8Z9_MESAU | Klkb1 | 1 | N | -- | -- | -- | -- |
| 115 | plasma protease C1 inhibitor | A0A1U8CDV4_MESAU | Serping1 | 1^PRM^ | N | -- | -- | -- | -- |
| 116 | plasminogen | A0A1U8CMG0_MESAU | Plg | 1^PRM^ | N | 1 | N | 1^PRM^ | R^a^ |
| 117 | platelet basic protein | A0A1U7QAS5_MESAU | Ppbp | 1^PRM^ | N | 1^PRM^ | R | 1 | R^a^ |
| 118 | Pregnancy protein | Q60552_MESAU | Hpp | -- | -- | 1^PRM^ | N | -- | -- |
| 119 | prenylcysteine oxidase 1 | A0A1U7QQY0_MESAU | Pcyox1 | 1^PRM^ | R^a^ | 1^PRM^ | R^a^ | 1^PRM^ | R^a^ |
| 120 | probable carboxypeptidase PM20D1 | A0A1U7R725_MESAU | Pm20d1 | 1 | N | -- | -- | -- | -- |
| 121 | profilin-1 | A0A1U7QJS1_MESAU | Pfn1 | 1^PRM^ | N | -- | -- | -- | -- |
| 122 | pro-opiomelanocortin | A0A1U7R1K9_MESAU | Pomc | 1 | N | -- | -- | -- | -- |
| 123 | properdin | A0A1U7R4J5_MESAU | Cfp | 1^PRM^ | N | 1 | N | -- | -- |
| 124 | proprotein convertase subtilisin/kexin type 9 | A0A1U8BW42_MESAU | Pcsk9 | 1^PRM^ | N | -- | -- | -- | -- |
| 125 | prosaposin | A0A1U8BPW9_MESAU | Psap | 1 | N | 1 | N | 1 | R^a^ |
| 126 | protein AMBP | A0A1U7QUT5_MESAU | Ambp | 1^PRM^ | R | -- | -- | 1 | R^a^ |
| 127 | protein FAM171B | A0A1U7QFT6_MESAU | Fam171b | 1 | N | -- | -- | -- | -- |
| 128 | protein LEG1 homolog | A0A1U7Q865_MESAU | LOC101843817 | -- | -- | -- | -- | 1 | N |
| 129 | prothrombin | A0A1U7QFC8_MESAU | F2 | 1^PRM^ | R | 1^PRM^ | R | 1 | R^a^ |
| 130 | pulmonary surfactant-associated protein B | A0A1U7QKZ9_MESAU | Sftpb | -- | -- | -- | -- | 1^PRM^ | R^a^ |
| 131 | retinoic acid receptor responder protein 2 | A0A1U8D1P0_MESAU | Rarres2 | 1 | N | -- | -- | -- | -- |
| 132 | retinol-binding protein 4 | A0A1U7QD37_MESAU | Rbp4 | 1 | N | -- | -- | 1 | R^a^ |
| 133 | rho GDP-dissociation inhibitor 2 | A0A1U8BXQ8_MESAU | Arhgdib | 1 | N | -- | -- | -- | -- |
| 134 | secreted phosphoprotein 24 | A0A1U7QZ59_MESAU | Spp2 | 1^PRM^ | N | -- | -- | -- | -- |
| 135 | serine protease inhibitor A3N | A0A1U8BVP4_MESAU | LOC101835835 | 1^PRM^ | N | 1^PRM^ | N | 1^PRM^ | N |
| 136 | serotransferrin | A0A1U8BSW4_MESAU | Tf | 1^PRM^ | N | 1^PRM^ | R | 1^PRM^ | R^a^ |
| 137 | serum albumin | A0A1U7Q2I1_MESAU | Alb | 1 | R^a^ | 1^PRM^ | R^a^ | 1^PRM^ | R^a^ |
| 138 | Serum amyloid A-1 protein | P20726_MESAU | SAA1 | 1^PRM^ | R^a^ | 1^PRM^ | R^a^ | -- | -- |
| 139 | Serum amyloid A-2 protein | P20727_MESAU | SAA2 | -- | -- | -- | -- | 1 | R^a^ |
| 140 | Serum amyloid A-3 protein | P19453_MESAU | SAA3 | 1 | N | 1 | N | 1 | N |
| 141 | Serum amyloid A-5 protein | P81491_MESAU | SAA5 | 1^PRM^ | N | 1^PRM^ | N | 1^PRM^ | N |
| 142 | serum amyloid P-component | A0A1U7QG20_MESAU | Apcs | 1 | N | -- | -- | -- | -- |
| 143 | serum paraoxonase/arylesterase 1 | A0A1U7RA96_MESAU | Pon1 | 1^PRM^ | R | 1^PRM^ | R | 1^PRM^ | R^a^ |
| 144 | tetranectin | A0A1U7QW95_MESAU | Clec3b, Tna | 1^PRM^ | N | -- | -- | -- | -- |
| 145 | thioredoxin | A0A1U7R0Q7_MESAU | trx | 1 | N | -- | -- | -- | -- |
| 146 | thrombospondin-1 isoform X1 | A0A1U7Q315_MESAU | Thbs1 | 1 | N | -- | -- | 1 | R^a^ |
| 147 | thymosin beta-10 | A0A1U8CFS1_MESAU | Tmsb10 | 1 | N | -- | -- | -- | -- |
| 148 | thymosin beta-4 | A0A1U7QT87_MESAU | Tmsb4x | 1^PRM^ | N | 1 | N | -- | -- |
| 149 | tissue factor pathway inhibitor isoform X2 | A0A1U7Q399 _MESAU | Tfpi | -- | -- | -- | -- | 1 | R |
| 150 | transthyretin | A0A1U7Q643_MESAU | Ttr | 1^PRM^ | N | 1^PRM^ | R | 1^PRM^ | R^a^ |
| 151 | trem-like transcript 1 protein | A0A1U7QHK8_MESAU | Treml1 | 1 | N | 1 | N | -- | -- |
| 152 | ubiquitin-40S ribosomal protein S27a | A0A1U7QM01_MESAU | Rps27a | 1 | N | -- | -- | 1 | N |
| 153 | Uteroglobin | Q8VD96_MESAU | SCGB1A1 | 1^PRM^ | N | -- | -- | -- | -- |
| 154 | vascular cell adhesion protein 1 | A0A1U7QQK8_MESAU | Vcam1 | -- | -- | -- | -- | 1 | N |
| 155 | vitamin D binding protein | A0A1U8BMS0_MESAU | Gc | 1^PRM^ | R^a^ | 1^PRM^ | N | 1 | R^a^ |
| 156 | vitronectin | A0A1U7QY02_MESAU | Vtn | 1^PRM^ | R | 1 | R | 1 | R^a^ |
| 157 | zinc-alpha-2-glycoprotein | A0A1U7R6L4_MESAU | Azgp1 | 1^PRM^ | N | -- | -- | -- | -- |

**R:** reported proteins referred to HDL/LDL/VLDL proteome list (Excel S1-1/2/3). **R ^a^:** likely proteins which detected in at least 3 different studies.

**N:** newly detected proteins in that lipoprotein. **N ^b^:** it's likely a contaminant. **1:** Proteins detected by label-free proteomics. **1^PRM^:** Proteins validated by PRM targeted proteomics.

**Table S2. Biological functions of the identified and validated proteins in VLDL, LDL, and HDL from golden hamsters**
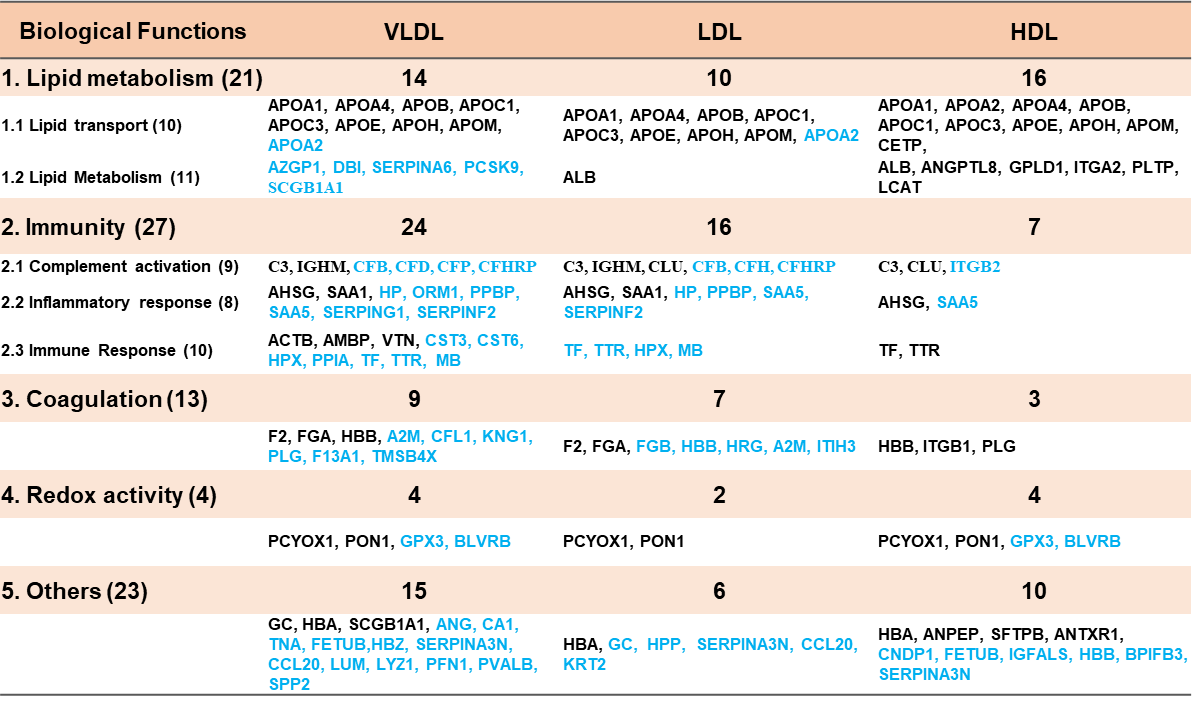


**Black color**: proteins were reported and validated in lipoprotein particles. **Blue color**: proteins were firstly detected and validated in the corresponding lipoprotein particles. The detailed description is in **Table S1 and Excel S2**.

**Table S3. Lipid profile of golden hamsters fed with normal or high-fat diets (Mean ± SD)**

| **Time** | **Indicators** | **Control (n = 11)** |  | **Model (n = 11)** |
| --- | --- | --- | --- | --- |
| 14^th^ week | TG (mmol/l) | 1.55 ± 0.23 |  | 2.87 ± 0.67 *** |
|  | TC (mmol/l) | 2.78 ± 0.35 |  | 4.26 ± 0.32 ** |
|  | LDL-C (mmol/l) | 0.72 ± 0.19 |  | 1.09 ± 0.13 * |
|  | HDL-C (mmol/l) | 1.54 ± 0.21 |  | 1.82 ± 0.29 * |

Model Group versus Control Group：** p<0.01，*** p<0.001

| **Table S4. Proteins identified in human serum three lipoproteins by label-free proteomics and validated by PRM targeted proteomics** | | | | | | | | | |  |
| --- | --- | --- | --- | --- | --- | --- | --- | --- | --- | --- |
| **No.** | **Protein name (134)** | **UniProt Accession*** | **Gene ID** | **VLDL** | | **LDL** | | **HDL** | | |
|  |  |  |  | **Our study** | **49 R ( 24 R^a^), 70 N (39 ^PRM^)** | **Our study** | **40 R ( 26 R^a^),**  **21 N (9 ^PRM^)** | **Our study** | **71 R ( 67 R^a^), 4 N (1 ^PRM^)** | |
| 1 | Actin, cytoplasmic 1 | A0A2R8Y793_HUMAN | ACTB | 1 | R^a^ | -- | -- | 1 | N | |
| 2 | Alpha-1-acid glycoprotein 1 | A1AG1_HUMAN | ORM1 | 1^PRM^ | N | -- | -- | 1^PRM^ | R^a^ | |
| 3 | Alpha-1-acid glycoprotein 2 | A1AG2_HUMAN | ORM2 | 1^PRM^ | N | -- | -- | 1^PRM^ | R^a^ | |
| 4 | Alpha-1-antichymotrypsin | AACT_HUMAN | SERPINA3 | 1^PRM^ | N | -- | -- | 1^PRM^ | R^a^ | |
| 5 | Alpha-1-antitrypsin | A1AT_HUMAN | SERPINA1 | 1^PRM^ | R^a^ | 1^PRM^ | R^a^ | 1^PRM^ | R^a^ | |
| 6 | Alpha-1B-glycoprotein | A1BG_HUMAN | A1BG | 1^PRM^ | R | -- | -- | 1^PRM^ | R^a^ | |
| 7 | Alpha-2-antiplasmin | A2AP_HUMAN | SERPINF2 | 1 | N | 1 | N | 1^PRM^ | R^a^ | |
| 8 | Alpha-2-HS-glycoprotein | C9JV77_HUMAN | AHSG | 1^PRM^ | R | 1^PRM^ | R | 1^PRM^ | R^a^ | |
| 9 | Alpha-2-macroglobulin | A2MG_HUMAN | A2M | 1^PRM^ | N | -- | -- | -- | -- | |
| 10 | Angiogenin | ANGI_HUMAN | ANG | 1 | N | -- | -- | -- | -- | |
| 11 | Angiotensinogen | ANGT_HUMAN | AGT | 1^PRM^ | R | -- | -- | 1^PRM^ | R^a^ | |
| 12 | Antithrombin-III | ANT3_HUMAN | SERPINC1 | -- | -- | -- | -- | 1^PRM^ | R^a^ | |
| 13 | Apolipoprotein A-I | APOA1_HUMAN | APOA1 | 1^PRM^ | R^a^ | 1^PRM^ | R^a^ | 1^PRM^ | R^a^ | |
| 14 | Apolipoprotein A-II | APOA2_HUMAN | APOA2 | 1^PRM^ | N | 1^PRM^ | R^a^ | 1^PRM^ | R^a^ | |
| 15 | Apolipoprotein A-IV | APOA4_HUMAN | APOA4 | 1^PRM^ | R^a^ | 1^PRM^ | R^a^ | 1^PRM^ | R^a^ | |
| 16 | Apolipoprotein A-V | APOA5_HUMAN | APOA5 | 1 | R | -- | -- | -- | -- | |
| 17 | Apolipoprotein B-100 | APOB_HUMAN | APOB | 1^PRM^ | R^a^ | 1^PRM^ | R^a^ | 1^PRM^ | R^a^ | |
| 18 | Apolipoprotein C-I | APOC1_HUMAN | APOC1 | 1^PRM^ | R^a^ | 1^PRM^ | R^a^ | 1^PRM^ | R^a^ | |
| 19 | Apolipoprotein C-II | APOC2_HUMAN | APOC2 | 1 | R^a^ | 1 | R^a^ | 1 | R^a^ | |
| 20 | Apolipoprotein C-III | APOC3_HUMAN | APOC3 | 1^PRM^ | R^a^ | 1^PRM^ | R^a^ | 1^PRM^ | R^a^ | |
| 21 | Apolipoprotein C-IV | APOC4_HUMAN | APOC4 | 1^PRM^ | R^a^ | 1^PRM^ | R^a^ | 1^PRM^ | R^a^ | |
| 22 | Apolipoprotein D | APOD_HUMAN | APOD | 1^PRM^ | R^a^ | 1^PRM^ | R^a^ | 1^PRM^ | R^a^ | |
| 23 | Apolipoprotein E | APOE_HUMAN | APOE | 1^PRM^ | R^a^ | 1^PRM^ | R^a^ | 1^PRM^ | R^a^ | |
| 24 | Apolipoprotein F | APOF_HUMAN | APOF | 1^PRM^ | R^a^ | 1^PRM^ | R^a^ | 1^PRM^ | R^a^ | |
| 25 | Apolipoprotein L1 | APOL1_HUMAN | APOL1 | 1^PRM^ | R | 1^PRM^ | R | 1^PRM^ | R^a^ | |
| 26 | Apolipoprotein M | Q5SRP5_HUMAN | APOM | 1 | R^a^ | 1^PRM^ | R^a^ | 1^PRM^ | R^a^ | |
| 27 | Apolipoprotein(a) | APOA_HUMAN | LPA | 1^PRM^ | R^a^ | 1^PRM^ | R^a^ | 1 | R^a^ | |
| 28 | Beta-2-glycoprotein 1 | APOH_HUMAN | APOH | 1^PRM^ | R | 1^PRM^ | R | 1^PRM^ | R^a^ | |
| 29 | Beta-2-microglobulin | B2MG_HUMAN | B2M | 1^PRM^ | N | -- | -- | -- | -- | |
| 30 | Brain acid soluble protein 1 | BASP1_HUMAN | BASP1 | 1 | N | -- | -- | -- | -- | |
| 31 | C4b-binding protein alpha chain | C4BPA_HUMAN | C4BPA | 1^PRM^ | R | -- | -- | -- | -- | |
| 32 | Calponin | A0A087X271_HUMAN | CNN2 | 1^PRM^ | N | -- | -- | -- | -- | |
| 33 | Cathelicidin antimicrobial peptide | CAMP_HUMAN | CAMP | -- | -- | 1^PRM^ | R^a^ | -- | -- | |
| 34 | CD5 antigen-like | CD5L_HUMAN | CD5L | 1 | N | -- | -- | -- | -- | |
| 35 | Cholesteryl ester transfer protein | CETP_HUMAN | CETP | 1^PRM^ | R | -- | -- | -- | -- | |
| 36 | Chromogranin-A | CMGA_HUMAN | CHGA | 1 | N | -- | -- | -- | -- | |
| 37 | Clusterin | CLUS_HUMAN | CLU | 1^PRM^ | R^a^ | 1^PRM^ | R^a^ | 1^PRM^ | R^a^ | |
| 38 | Coagulation factor V | A0A0A0MRJ7_HUMAN | F5 | 1^PRM^ | N | -- | -- | -- | -- | |
| 39 | Cofilin-1 | COF1_HUMAN | CFL1 | 1^PRM^ | N | -- | -- | -- | -- | |
| 40 | Coiled-coil domain-containing protein 40 | CCD40_HUMAN | CCDC40 | 1 | N | -- | -- | -- | -- | |
| 41 | Collagen alpha-1(I) chain | CO1A1_HUMAN | COL1A1 | 1^PRM^ | N | -- | -- | -- | -- | |
| 42 | Complement C3 | CO3_HUMAN | C3 | 1^PRM^ | R^a^ | 1^PRM^ | R^a^ | 1^PRM^ | R^a^ | |
| 43 | Complement C4-B | A0A0G2JL54_HUMAN | C4B | 1^PRM^ | R^a^ | 1^PRM^ | R^a^ | 1^PRM^ | R^a^ | |
| 44 | Complement component C9 | CO9_HUMAN | C9 | 1^PRM^ | R | 1^PRM^ | N | 1^PRM^ | R^a^ | |
| 45 | Complement factor B | A0A0G2JH38_HUMAN | CFB | -- | -- | -- | -- | 1^PRM^ | R^a^ | |
| 46 | Complement factor D | CFAD_HUMAN | CFD | -- | -- | -- | -- | 1^PRM^ | R^a^ | |
| 47 | Complement factor H-related protein 4 | FHR4_HUMAN | CFHR4 | 1^PRM^ | R | -- | -- | -- | -- | |
| 48 | C-reactive protein | CRP_HUMAN | CRP | 1^PRM^ | N | -- | -- | -- | -- | |
| 49 | Cystatin-C | CYTC_HUMAN | CST3 | 1^PRM^ | N | 1 | R | -- | -- | |
| 50 | Cysteine and glycine-rich protein 1 | CSRP1_HUMAN | CSRP1 | 1 | N | -- | -- | -- | -- | |
| 51 | Fermitin family homolog 3 | URP2_HUMAN | FERMT3 | 1^PRM^ | N | -- | -- | -- | -- | |
| 52 | Fibrinogen alpha chain | FIBA_HUMAN | FGA | 1^PRM^ | R^a^ | 1 | R^a^ | 1 | R^a^ | |
| 53 | Fibronectin | FINC_HUMAN | FN1 | 1 | N | -- | -- | -- | -- | |
| 54 | Fibulin-1 | FBLN1_HUMAN | FBLN1 | 1 | N | -- | -- | -- | -- | |
| 55 | Filamin-A | FLNA_HUMAN | FLNA | 1^PRM^ | N | -- | -- | -- | -- | |
| 56 | Gelsolin | A0A0A0MS51_HUMAN | GSN | 1 | N | -- | -- | 1^PRM^ | R^a^ | |
| 57 | Glutathione peroxidase | A0A087X1J7_HUMAN | GPX3 | -- | -- | -- | -- | 1^PRM^ | N | |
| 58 | Haptoglobin | HPT_HUMAN | HP | 1^PRM^ | N | -- | -- | 1^PRM^ | R^a^ | |
| 59 | Haptoglobin-related protein | HPTR_HUMAN | HPR | 1^PRM^ | R^a^ | 1 | R^a^ | 1^PRM^ | R^a^ | |
| 60 | Hemoglobin subunit alpha | A0A2R8Y7C0_HUMAN | HBA2 | 1^PRM^ | R | -- | -- | 1^PRM^ | R^a^ | |
| 61 | Hemoglobin subunit beta | HBB_HUMAN | HBB | 1^PRM^ | R | 1^PRM^ | R | 1^PRM^ | R^a^ | |
| 62 | Hemopexin | HEMO_HUMAN | HPX | 1^PRM^ | N | 1^PRM^ | N | 1^PRM^ | R^a^ | |
| 63 | Heparin cofactor 2 | HEP2_HUMAN | SERPIND1 | 1^PRM^ | N | -- | -- | 1^PRM^ | R^a^ | |
| 64 | Immunoglobulin heavy constant alpha 1 | A0A286YEY1_HUMAN | IGHA1 | 1^PRM^ | R^a^ | 1^PRM^ | R^a^ | 1^PRM^ | R^a^ | |
| 65 | Immunoglobulin heavy constant gamma 1 | A0A0A0MS08_HUMAN | IGHG1 | 1^PRM^ | R | 1^PRM^ | R | 1^PRM^ | R^a^ | |
| 66 | Immunoglobulin heavy constant gamma 2 | A0A286YEY4_HUMAN | IGHG2 | 1^PRM^ | R | 1 | N | 1^PRM^ | R^a^ | |
| 67 | Immunoglobulin heavy constant gamma 3 | A0A286YES1_HUMAN | IGHG3 | 1^PRM^ | N | 1 | N | -- | -- | |
| 68 | Immunoglobulin heavy constant mu | A0A1B0GUU9_HUMAN | IGHM | 1^PRM^ | R | 1^PRM^ | N | 1^PRM^ | R^a^ | |
| 69 | Immunoglobulin heavy variable 3-74 | HV374_HUMAN | IGHV3-74 | 1 | N | -- | -- | -- | -- | |
| 70 | Immunoglobulin J chain | D6RD17_HUMAN | JCHAIN | 1^PRM^ | N | -- | -- | -- | -- | |
| 71 | Ig kappa chain C region | IGKC_HUMAN | IGKC | 1^PRM^ | R | 1^PRM^ | R^a^ | 1^PRM^ | R^a^ | |
| 72 | Immunoglobulin lambda constant 2 | IGLC2_HUMAN | IGLC2 | 1 | N | -- | -- | -- | -- | |
| 73 | Insulin-like growth factor-binding protein 2 | IBP2_HUMAN | IGFBP2 | 1^PRM^ | N | -- | -- | -- | -- | |
| 74 | Insulin-like growth factor-binding protein 3 | A6XND0_HUMAN | IGFBP3 | 1 | N | -- | -- | -- | -- | |
| 75 | Insulin-like growth factor-binding protein 4 | IBP4_HUMAN | IGFBP4 | 1^PRM^ | N | -- | -- | -- | -- | |
| 76 | Inter-alpha-trypsin inhibitor heavy chain H2 | ITIH2_HUMAN | ITIH2 | 1^PRM^ | N | 1^PRM^ | N | -- | -- | |
| 77 | Inter-alpha-trypsin inhibitor heavy chain H4 | ITIH4_HUMAN | ITIH4 | 1^PRM^ | R | 1^PRM^ | R | 1^PRM^ | R^a^ | |
| 78 | Keratin, type I cuticular Ha3-II | KT33B_HUMAN | KRT33B | 1 | N^b^ | -- | -- | -- | -- | |
| 79 | Keratin, type I cytoskeletal 10 | K1C10_HUMAN | KRT10 | 1^PRM^ | N^b^ | 1^PRM^ | N^b^ | -- | -- | |
| 80 | Keratin, type I cytoskeletal 9 | K1C9_HUMAN | KRT9 | 1^PRM^ | N^b^ | -- | -- | -- | -- | |
| 81 | Keratin, type II cuticular Hb3 | KRT83_HUMAN | KRT83 | 1 | N^b^ | -- | -- | -- | -- | |
| 82 | Keratin, type II cytoskeletal 1 | K2C1_HUMAN | KRT1 | 1^PRM^ | N^b^ | 1 | N^b^ | 1^PRM^ | R | |
| 83 | Keratin, type II cytoskeletal 2 epidermal | K22E_HUMAN | KRT2 | 1^PRM^ | N^b^ | -- | -- | -- | -- | |
| 84 | Kininogen-1 | KNG1_HUMAN | KNG1 | 1^PRM^ | N | 1^PRM^ | N | 1^PRM^ | R^a^ | |
| 85 | Leucine-rich alpha-2-glycoprotein | A2GL_HUMAN | LRG1 | -- | -- | -- | -- | 1^PRM^ | R^a^ | |
| 86 | Lipopolysaccharide-binding protein | LBP_HUMAN | LBP | 1^PRM^ | R | 1^PRM^ | N | 1^PRM^ | R^a^ | |
| 87 | Low-density lipoprotein receptor | H0YMD1_HUMAN | LDLR | 1^PRM^ | R | -- | -- | -- | -- | |
| 88 | Myotrophin | MTPN_HUMAN | MTPN | 1 | N | -- | -- | -- | -- | |
| 89 | Nesprin-1 | A0A0C4DG40_HUMAN | SYNE1 | -- | -- | 1 | N | -- | -- | |
| 90 | Neuroblast differentiation-associated protein AHNAK | AHNK_HUMAN | AHNAK | 1 | N | -- | -- | -- | -- | |
| 91 | Neutrophil defensin 1 | DEF1_HUMAN | DEFA1 | 1 | N | -- | -- | 1 | N | |
| 92 | Nicotinate phosphoribosyltransferase | H0YDA6_HUMAN | NAPRT | -- | -- | 1 | N | -- | -- | |
| 93 | Nucleolar transcription factor 1 | E9PKP7_HUMAN | UBTF | 1 | N | -- | -- | -- | -- | |
| 94 | Otoferlin | OTOF_HUMAN | OTOF | 1 | N | -- | -- | -- | -- | |
| 95 | PDZ and LIM domain protein 1 | PDLI1_HUMAN | PDLIM1 | 1^PRM^ | N | -- | -- | -- | -- | |
| 96 | Peptidyl-prolyl cis-trans isomerase A | PPIA_HUMAN | PPIA | 1 | N | -- | -- | -- | -- | |
| 97 | Phosphatidylcholine-sterol acyltransferase | LCAT_HUMAN | LCAT | -- | -- | -- | -- | 1^PRM^ | R^a^ | |
| 98 | Phospholipid transfer protein | PLTP_HUMAN | PLTP | 1 | R | -- | -- | 1^PRM^ | R^a^ | |
| 99 | Pigment epithelium-derived factor | PEDF_HUMAN | SERPINF1 | -- | -- | -- | -- | 1^PRM^ | R^a^ | |
| 100 | Plasma protease C1 inhibitor | E9PGN7_HUMAN | SERPING1 | 1 | N | -- | -- | 1^PRM^ | R^a^ | |
| 101 | Plasminogen | PLMN_HUMAN | PLG | 1 | N | 1 | N | -- | -- | |
| 102 | Platelet basic protein | CXCL7_HUMAN | PPBP | 1^PRM^ | N | 1^PRM^ | R | 1^PRM^ | R^a^ | |
| 103 | Platelet factor 4 | PLF4_HUMAN | PF4 | 1^PRM^ | N | 1^PRM^ | R | -- | -- | |
| 104 | Platelet-activating factor acetylhydrolase | PAFA_HUMAN | PLA2G7 | -- | -- | 1^PRM^ | R | -- | -- | |
| 105 | Polyubiquitin-B | B4DV12_HUMAN | UBB | 1 | N | -- | -- | -- | -- | |
| 106 | POTE ankyrin domain family member I | POTEI_HUMAN | POTEI | -- | -- | 1 | N | -- | -- | |
| 107 | Prenylcysteine oxidase 1 | PCYOX_HUMAN | PCYOX1 | 1^PRM^ | R^a^ | 1^PRM^ | R^a^ | 1^PRM^ | R^a^ | |
| 108 | Profilin-1 | PROF1_HUMAN | PFN1 | 1^PRM^ | N | -- | -- | 1 | R | |
| 109 | Protein AMBP | AMBP_HUMAN | AMBP | 1^PRM^ | R | 1^PRM^ | N | 1^PRM^ | R^a^ | |
| 110 | Protein MENT | MENT_HUMAN | MENT | 1^PRM^ | N | -- | -- | 1^PRM^ | R | |
| 111 | Protein/nucleic acid deglycase DJ-1 | K7ELW0_HUMAN | PARK7 | 1 | N | -- | -- | -- | -- | |
| 112 | Prothrombin | E9PIT3_HUMAN | F2 | 1^PRM^ | R | 1^PRM^ | R | 1^PRM^ | R^a^ | |
| 113 | Pulmonary surfactant-associated protein B | D6W5L6_HUMAN | SFTPB | -- | -- | -- | -- | 1^PRM^ | R^a^ | |
| 114 | Retinol-binding protein | Q5VY30_HUMAN | RBP4 | 1^PRM^ | N | 1 | N | 1^PRM^ | R^a^ | |
| 115 | Rho GDP-dissociation inhibitor 2 | F5H2R5_HUMAN | ARHGDIB | 1 | N | -- | -- | -- | -- | |
| 116 | Ribonuclease 4 | RNAS4_HUMAN | RNASE4 | 1^PRM^ | N | -- | -- | -- | -- | |
| 117 | SAA2-SAA4 readthrough | A0A096LPE2_HUMAN | SAA2-SAA4 | 1 | N | 1 | N | 1 | N | |
| 118 | Serotransferrin | TRFE_HUMAN | TF | 1^PRM^ | N | 1^PRM^ | R | 1^PRM^ | R^a^ | |
| 119 | Serum albumin | ALBU_HUMAN | ALB | 1^PRM^ | R^a^ | 1^PRM^ | R^a^ | 1^PRM^ | R^a^ | |
| 120 | Serum amyloid A-1 protein | SAA1_HUMAN | SAA1 | 1^PRM^ | R^a^ | 1^PRM^ | R^a^ | 1^PRM^ | R^a^ | |
| 121 | Serum amyloid A-2 protein | SAA2_HUMAN | SAA2 | 1^PRM^ | R^a^ | 1^PRM^ | R^a^ | 1^PRM^ | R^a^ | |
| 122 | Serum paraoxonase/arylesterase 1 | PON1_HUMAN | PON1 | 1^PRM^ | R | 1^PRM^ | R | 1^PRM^ | R^a^ | |
| 123 | Serum paraoxonase/lactonase 3 | PON3_HUMAN | PON3 | -- | -- | -- | -- | 1^PRM^ | R^a^ | |
| 124 | SH3 domain-binding glutamic acid-rich-like protein 3 | Q5T123_HUMAN | SH3BGRL3 | 1 | N | -- | -- | -- | -- | |
| 125 | Talin-1 | TLN1_HUMAN | TLN1 | 1^PRM^ | N | 1 | N | 1^PRM^ | R | |
| 126 | Thioredoxin | THIO_HUMAN | TXN | 1^PRM^ | N | -- | -- | -- | -- | |
| 127 | Thrombospondin-1 | TSP1_HUMAN | THBS1 | 1 | N | -- | -- | -- | -- | |
| 128 | Transgelin-2 | TAGL2_HUMAN | TAGLN2 | 1^PRM^ | N | -- | -- | -- | -- | |
| 129 | Trans-Golgi network integral membrane protein 2 | TGON2_HUMAN | TGOLN2 | 1 | N | -- | -- | -- | -- | |
| 130 | Transthyretin | A0A087WT59_HUMAN | TTR | -- | -- | -- | -- | 1^PRM^ | R^a^ | |
| 131 | Tropomyosin alpha-4 chain | TPM4_HUMAN | TPM4 | 1 | N | 1^PRM^ | N | -- | -- | |
| 132 | Vitamin D-binding protein | D6RF35_HUMAN | GC, VTDB | 1^PRM^ | R | 1 | N | 1^PRM^ | R^a^ | |
| 133 | Vitronectin | VTNC_HUMAN | VTN | 1^PRM^ | R | 1^PRM^ | R | 1^PRM^ | R^a^ | |
| 134 | Zinc-alpha-2-glycoprotein | ZA2G_HUMAN | AZGP1 | 1^PRM^ | N | -- | -- | 1^PRM^ | R^a^ | |

**R:** reported proteins referred to HDL/LDL/VLDL proteome list (Excel S1-1/2/3). **R ^a^:** likely proteins which detected in at least 3 different labs. **N:** newly detected proteins in that lipoprotein. **N ^b^:** it's likely a contaminant. **1:** Proteins detected by label-free proteomics. **1^PRM^:** Proteins validated by PRM targeted proteomics

**Table S5. The validated differential proteins link to metabolic diseases**

| **Particles** | **Differential  Proteins** | **Change  trend** | **Related to diseases with the same tendency of change** |
| --- | --- | --- | --- |
| VLDL | APOA4 | Up | ASCAD ^9^, CAD ^9^ |
|  | APOC3 | Up | ASCAD ^9^, CAD ^9^, CVD ^10^, type 2 diabete ^11^, hypertriglyceridemia ^12^, |
|  | CFB | Up | familial combined hyperlipidemia ^13^ |
|  | CFD | Up | obesity ^14^ |
|  | KNG1 | Up | hyperlipidemia ^15^ |
|  | SERPING1 | Up | hyperlipidemia (liver) ^16^ |
|  | A2M | Down | NAFLD (liver) ^17^ |
|  | FGA | Down | atherosclerosis ^18^ |
|  | HPX | Down | hyperlipidemia (Increased) ^19,20^, CHD ^19^ |
|  | ORM1 | Down |  |
|  | SERPINF2 | Down |  |
|  | VTN | Down |  |
| LDL | APOB | Up | ASCAD ^9^, CVD ^10,21^, Obesity ^22^, hyperlipidemia ^23^, |
|  | APOE | Up | ASCAD ^9^, CAD ^9^, CVD ^10^, type 2 diabetes ^11^, hyperlipidemia ^23^, NAFLD (liver) ^17^, atherosclerosis ^24^, |
|  | C3 | Up | Familial combined hyperlipidemia ^13^ |
|  | PPBP | Up | CHD ^25^ |
|  | SAA1 | Up | obesity ^26^, atherosclerosis ^27^, CVD ^28^ |
|  | HP | Down | hyperlipidemia ^19^ |
| HDL | APOA1 | Up | CVD ^10^, CAD ^9^, obesity ^22^, |
|  | APOA2 | Up | CAD ^9^ |
|  | APOM | Up | type 2 diabetes ^29^ |
|  | LCAT | Up | atherosclerosis ^30^, type 2 diabetes ^31^ |
|  | PCYOX1 | Up |  |
|  | PLTP | Up | CVD ^21^ |
|  | APOC1 | Down | obesity ^32^ |
|  | GPX3 | Down | atherosclerosis ^33^ |
|  | PON1 | Down | hyperlipidemia ^34^, atherosclerosis ^35^ |

**CVD:** cardiovascular disease; **CAD:** coronary artery disease; **ASCAD:** atherosclerotic cardiovascular disease;

**NAFLD:** non-alcoholic fatty liver disease; **CHD:** coronary heart disease; **CLD:** chronic liver diseases

# Figures


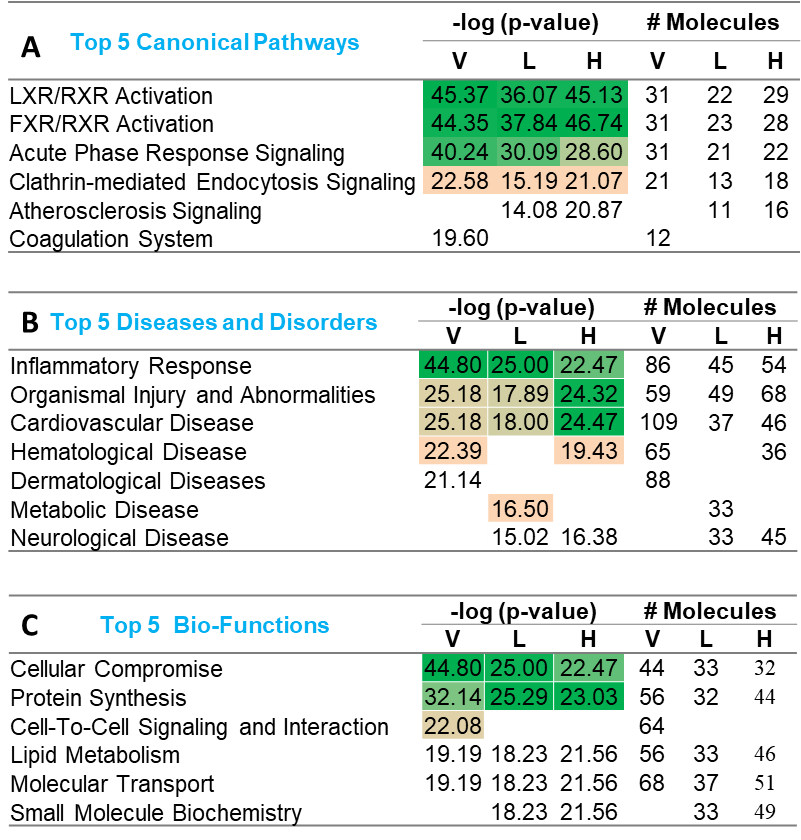


**Fig. S1. Protein functions of VLDL/LDL/HDL of golden hamsters by IPA software.**

Top 5 canonical pathways, diseases and disorders, bio-functions of identified proteins in VLDL/LDL/HDL from golden hamsters using the IPA software.


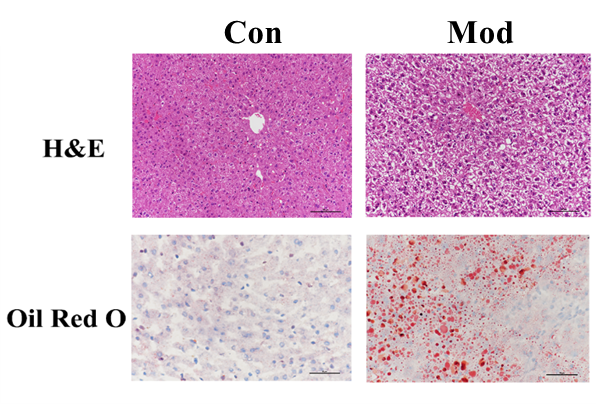


**Fig. S2. Liver sections were stained with hematoxylin and eosin (H&E) or oil red O and visualized.** (In this study, all samples of normal and hyperlipidemic golden hamsters were a part of our previous studies ^36^, <https://doi.org/10.1021/acs.analchem.8b05337>.


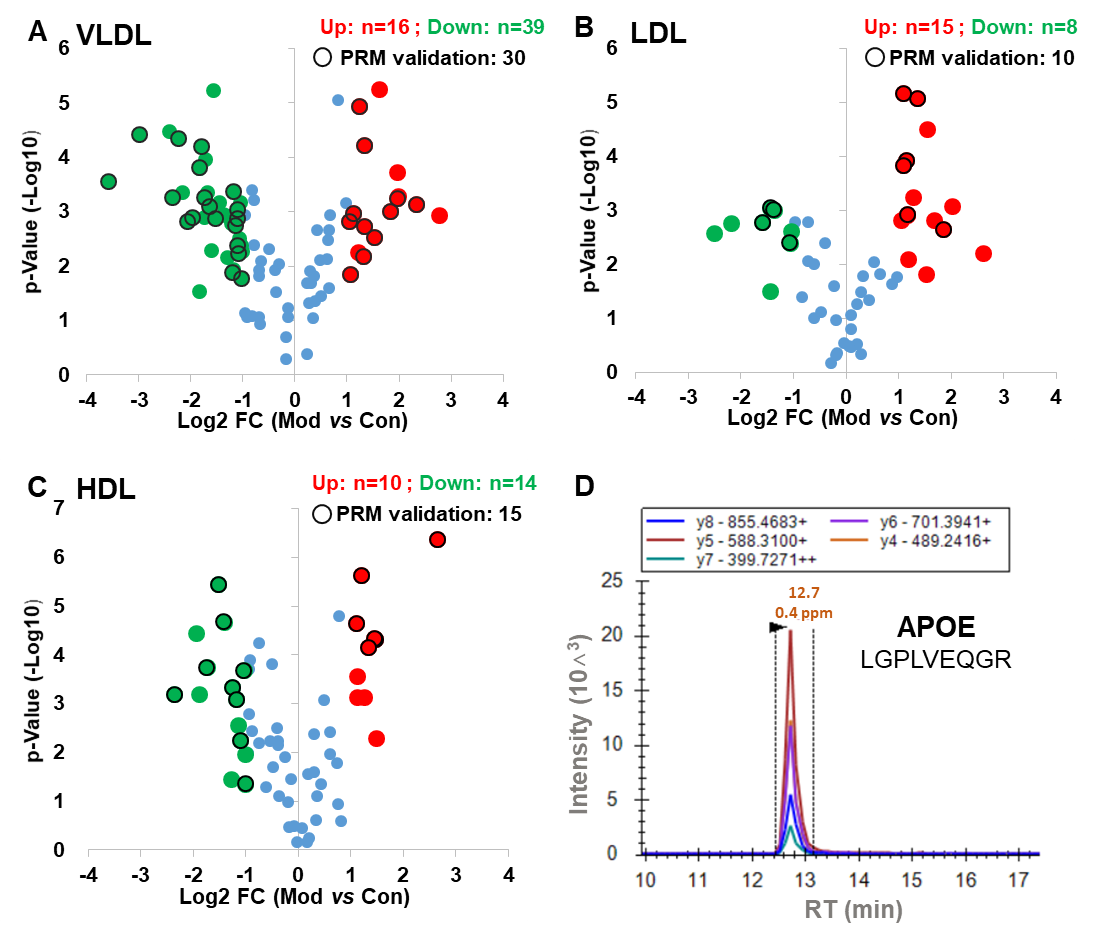


**Fig. S3. Volcano plot of identified proteins and representative transitions for PRM. (A-C)** Volcano plot of all proteins identified in VLDL, LDL, and HDL of normal and hyperlipidemic golden hamsters. The red and green dots display the remarkably upregulated and downregulated proteins in the Mod group compared with the Con group, respectively (*p*-value ≤ 0.05 and fold change ≥ 2). The black-bordered dots present the validated proteins with the same upregulated or downregulated trends. **(D)** Representative transitions of APOE in PRM targeted verification.


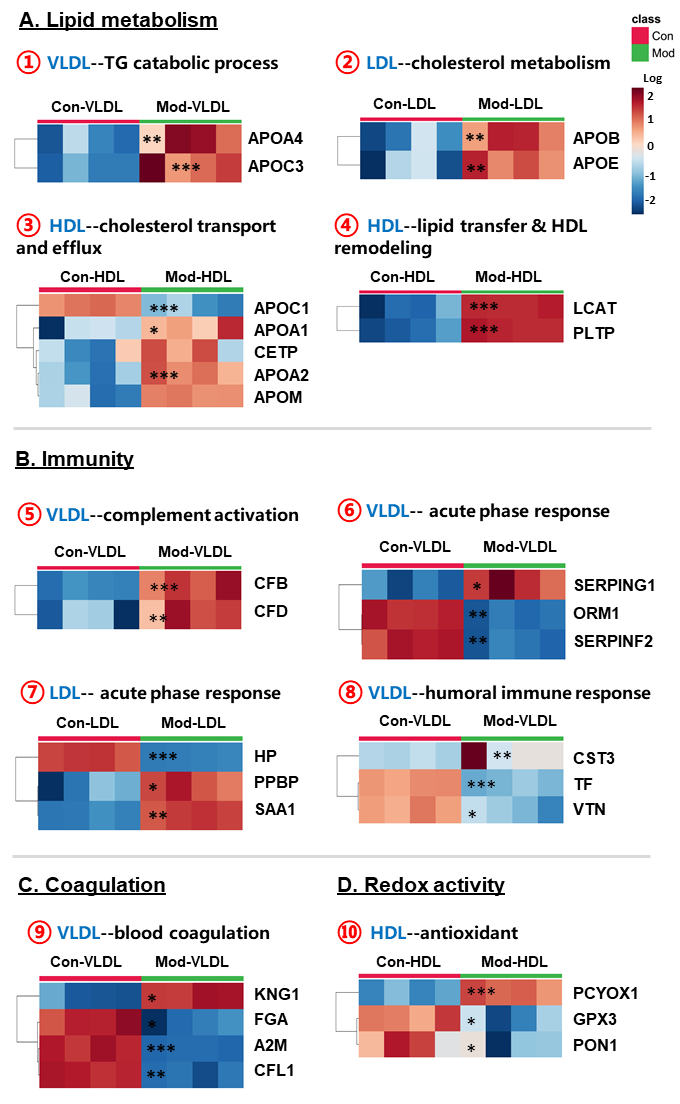


**Fig. S4. Heatmaps depicting the changes of** [**differential**](javascript:;) **proteins from normal and hyperlipidemic golden hamsters. (A)** Lipid metabolism; **(B)** immunity; **(C)** Coagulation; **(D)** Redox activity. [Differential](javascript:;) proteins were validated by PRM proteomics, which were also overlapped in corresponding lipoprotein particles between humans and golden hamsters.

*: p < 0.05, **: p < 0.01, ***: p < 0.001.


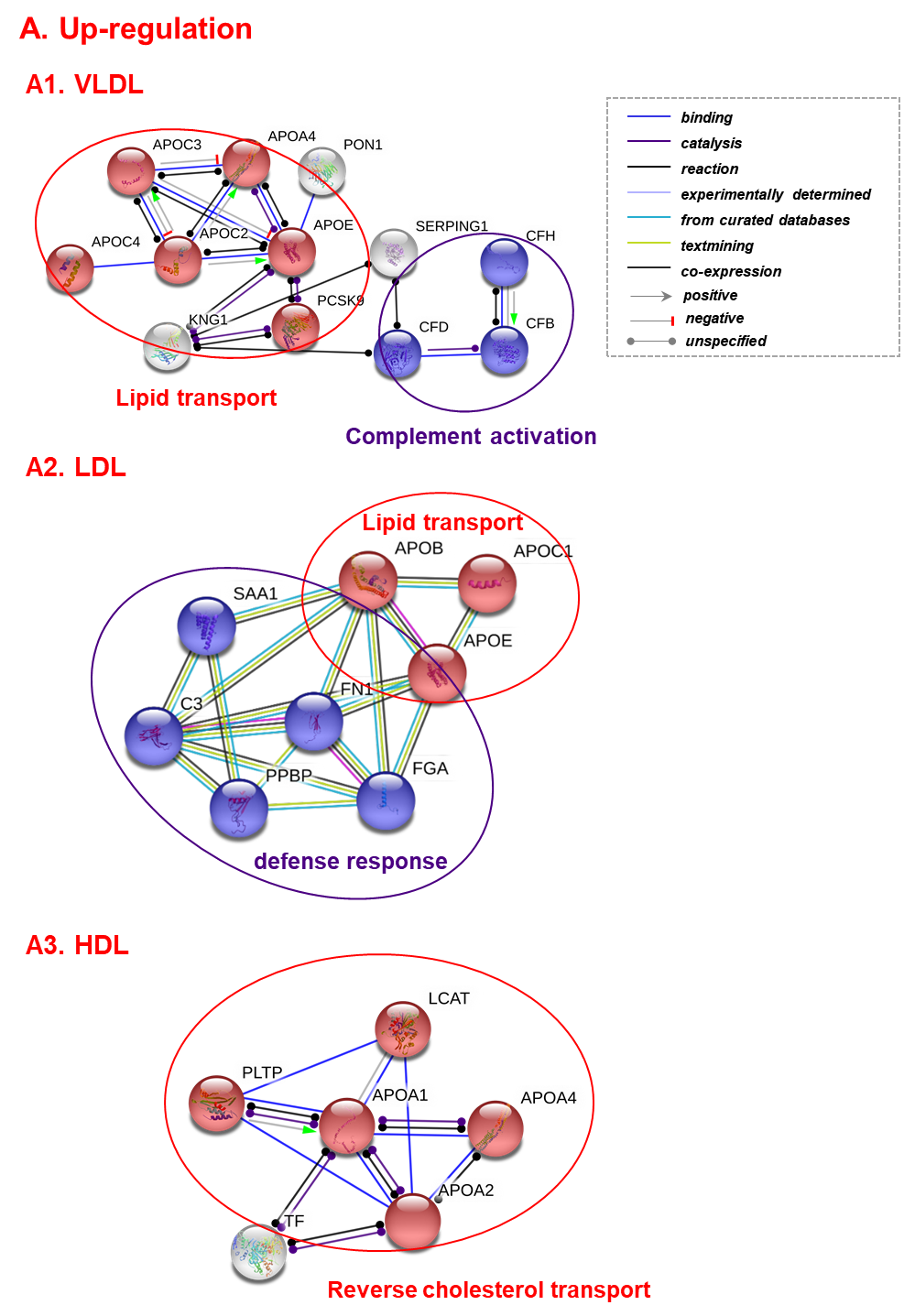


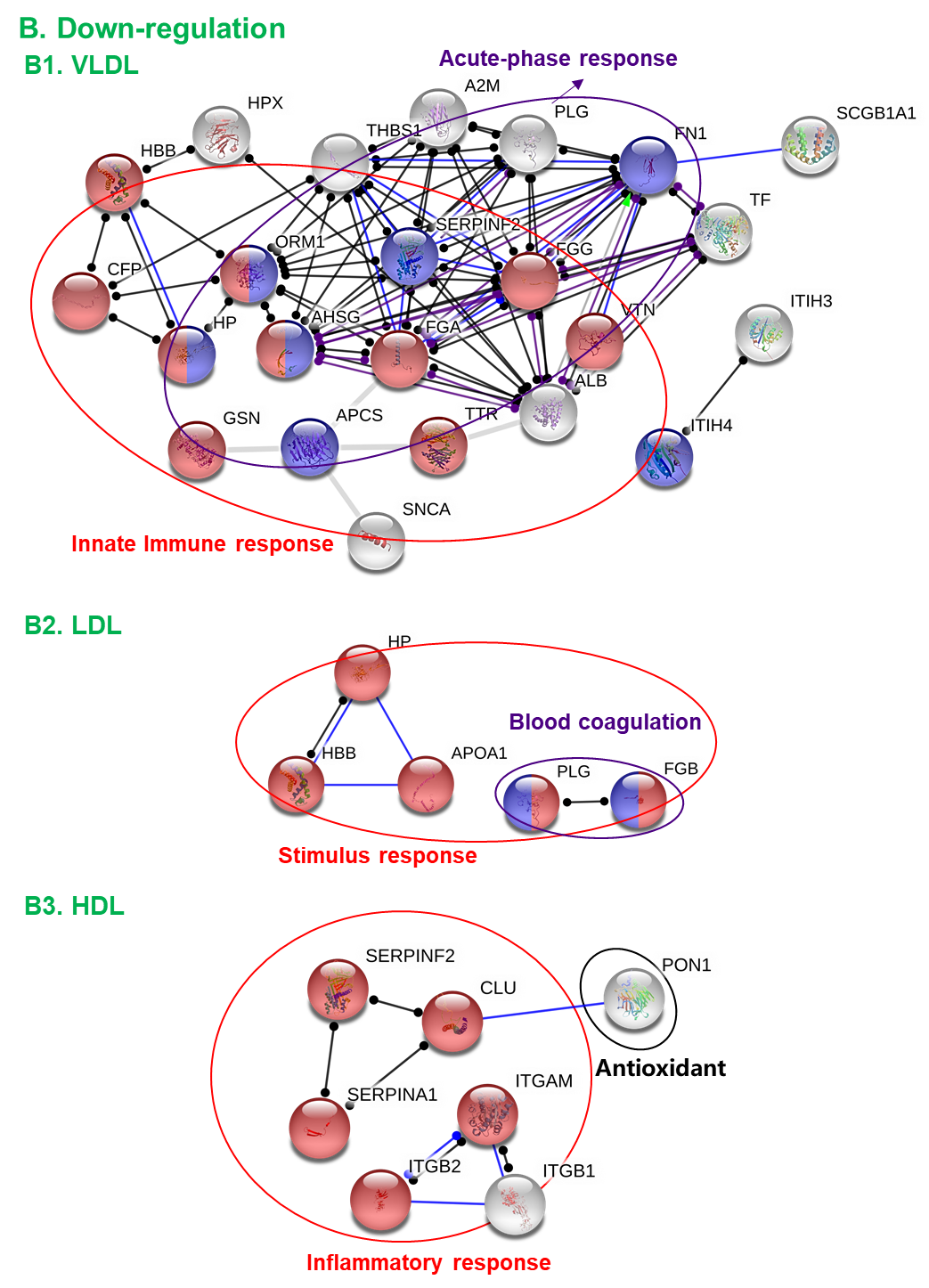


**Fig. S5. Interaction networks of differential proteins analyzed by the STRING database.**

**(A)** Networks of up-regulated proteins in VLDL, LDL, and HDL of hyperlipidemic hamsters compared to normal hamsters. **(B)** Networks of down-regulated proteins in VLDL, LDL, and HDL of hyperlipidemic hamsters compared to normal hamsters. Active interaction sources were from experiments, databases, and textmining. The minimum confidence of interaction score was set 0.900.


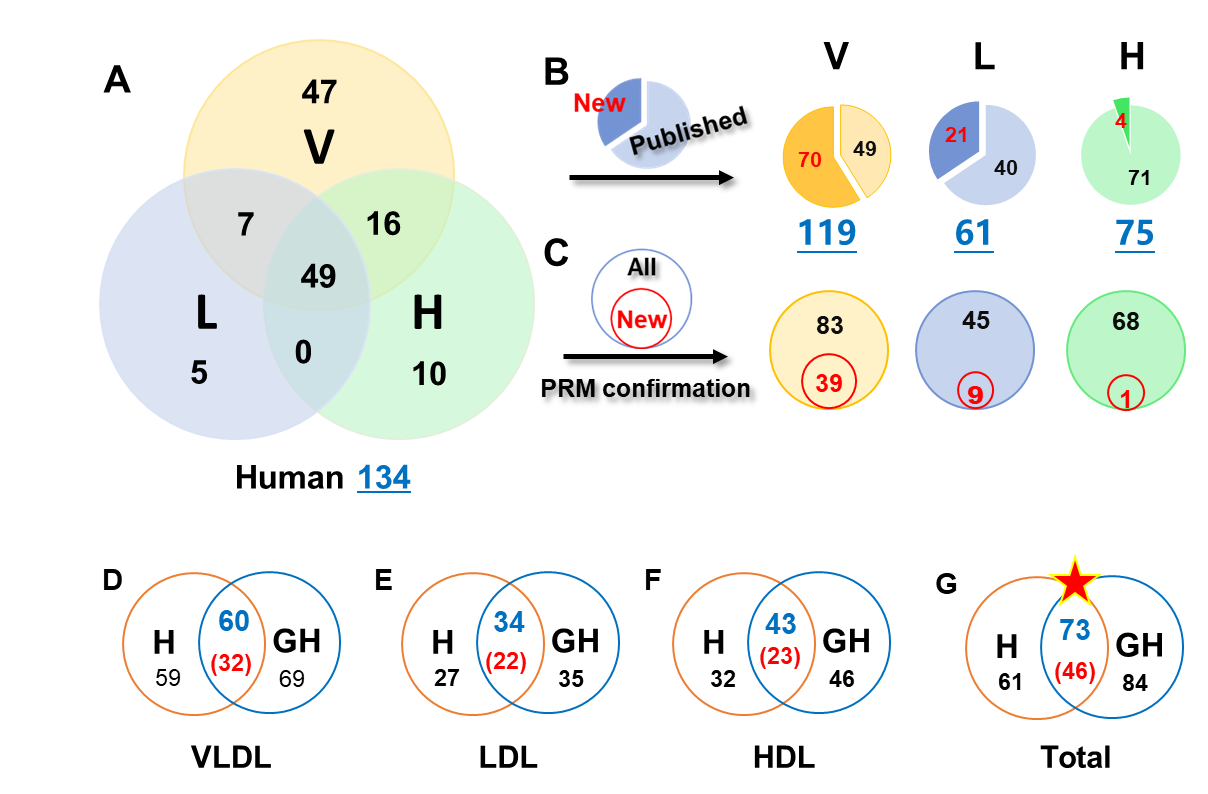


**Fig. S6. Protein overlaps in VLDL/LDL/HDL of humans and golden hamsters.**

**(A)** Venn diagram of identified protein in VLDL (V), LDL (L), and HDL (H) particles of humans. **(B)** Numbers of published (black) or newly detected (red) proteins compared with the VLDL/LDL/HDL proteome lists. **(C)** Numbers of validated proteins by PRM proteomics. Black: total numbers; red: number of new proteins. Unique peptide details listed in **Excel S3**. **(D-G)** Numbers of proteins overlapped in VLDL, LDL, HDL, and the total number of the three lipoproteins between humans (H) and golden hamsters (GH). Blue: total numbers; red: numbers of shared proteins confirmed by PRM analysis.

# Reference

1. Kailemia MJ, Wei W, Nguyen K, Beals E, Sawrey-Kubicek L, Rhodes C, Zhu C, Sacchi R, Zivkovic AM, Lebrilla CB. Targeted Measurements of O- and N-Glycopeptides Show That Proteins in High Density Lipoprotein Particles Are Enriched with Specific Glycosylation Compared to Plasma. *J Proteome Res*. Published online 2018. doi:10.1021/acs.jproteome.7b00604

2. Zhu C, Wong M, Li Q, Sawrey-Kubicek L, Beals E, Rhodes CH, Sacchi R, Lebrilla CB, Zivkovic AM. Site-Specific Glycoprofiles of HDL-Associated ApoE are Correlated with HDL Functional Capacity and Unaffected by Short-Term Diet. *J Proteome Res*. Published online 2019. doi:10.1021/acs.jproteome.9b00450

3. Wiśniewski JR, Zougman A, Nagaraj N, Mann M. Universal sample preparation method for proteome analysis. *Nat Methods*. Published online 2009. doi:10.1038/nmeth.1322

4. Ma J, Chen T, Wu S, Yang C, Bai M, Shu K, Li K, Zhang G, Jin Z, He F, Hermjakob H, Zhu Y. Iprox: An integrated proteome resource. *Nucleic Acids Res*. Published online 2019. doi:10.1093/nar/gky869

5. Keller A, Nesvizhskii AI, Kolker E, Aebersold R. Empirical statistical model to estimate the accuracy of peptide identifications made by MS/MS and database search. *Anal Chem*. 2002;74(20):5383-5392. doi:10.1021/ac025747h

6. Parker SS, Krantz J, Kwak EA, Barker NK, Deer CG, Lee NY, Mouneimne G, Langlais PR. Insulin induces microtubule stabilization and regulates the microtubule plus-end tracking protein network in adipocytes. *Mol Cell Proteomics*. Published online 2019. doi:10.1074/mcp.RA119.001450

7. Chen C, Chen H, He Y, Xia R. TBtools, a Toolkit for Biologists integrating various biological data handling tools with a user-friendly interface. *bioRxiv*. Published online 2018. doi:10.1101/289660

8. Henderson CM, Shulman NJ, MacLean B, MacCoss MJ, Hoofnagle AN. Skyline performs as well as vendor software in the quantitative analysis of serum 25-hydroxy Vitamin D and Vitamin D binding globulin. *Clin Chem*. Published online 2018. doi:10.1373/clinchem.2017.282293

9. Dittrich J, Beutner F, Teren A, Thiery J, Burkhardt R, Scholz M, Ceglarek U. Plasma levels of apolipoproteins C-III, A-IV, and E are independently associated with stable atherosclerotic cardiovascular disease. *Atherosclerosis*. Published online 2019. doi:10.1016/j.atherosclerosis.2018.11.006

10. Pechlaner R, Tsimikas S, Yin X, Willeit P, Baig F, Santer P, Oberhollenzer F, Egger G, Witztum JL, Alexander VJ, Willeit J, Kiechl S, Mayr M. Very-Low-Density Lipoprotein–Associated Apolipoproteins Predict Cardiovascular Events and Are Lowered by Inhibition of APOC-III. *J Am Coll Cardiol*. 2017;69(7):789-800. doi:10.1016/j.jacc.2016.11.065

11. Dallinga-Thie GM, Van Tol A, Hattori H, Van Vark-Van Der Zee LC, Jansen H, Sijbrands EJG. Plasma apolipoprotein A5 and triglycerides in type 2 diabetes. *Diabetologia*. Published online 2006. doi:10.1007/s00125-006-0261-0

12. Gaudet D, Alexander VJ, Baker BF, Brisson D, Tremblay K, Singleton W, Geary RS, Hughes SG, Viney NJ, Graham MJ, Crooke RM, Witztum JL, Brunzell JD, Kastelein JJP. Antisense inhibition of apolipoprotein C-III in patients with hypertriglyceridemia. *N Engl J Med*. Published online 2015. doi:10.1056/NEJMoa1400283

13. Ylitalo K, Porkka KVK, Meri S, Nuotio I, Suurinkeroinen L, Vakkilainen J, Pajukanta P, Viikari JSA, Peltonen L, Ehnholm C, Taskinen MR. Serum complement and familial combined hyperlipidemia. *Atherosclerosis*. Published online 1997. doi:10.1016/S0021-9150(96)06054-6

14. Rosen BS, Cook KS, Yaglom J, Groves DL, Volanakis JE, Damm D, White T, Spiegelman BM. Adipsin and complement factor D activity: An immune-related defect in obesity. *Science (80- )*. Published online 1989. doi:10.1126/science.2734615

15. Zhang Z, Wang W, Jin L, Cao X, Jian G, Wu N, Xu X, Yao Y, Wang D. ITRAQ-Based Quantitative Proteomics Analysis of the Protective Effect of Yinchenwuling Powder on Hyperlipidemic Rats. *Evidence-based Complement Altern Med*. Published online 2017. doi:10.1155/2017/3275096

16. Režen T, Tamasi V, Lövgren-Sandblom A, Björkhem I, Meyer UA, Rozman D. Effect of CAR activation on selected metabolic pathways in normal and hyperlipidemic mouse livers. *BMC Genomics*. Published online 2009. doi:10.1186/1471-2164-10-384

17. Liu Y, Yu F, Han Y, Li Q, Cao Z, Xiang X, Jiang S, Wang X, Lu J, Lai R, Wang H, Cai W, Bao S, Xie Q. SUMO-specific protease 3 is a key regulator for hepatic lipid metabolism in non-alcoholic fatty liver disease. *Sci Rep*. Published online 2016. doi:10.1038/srep37351

18. Parastatidis I, Thomson L, Fries DM, Moore RE, Tohyama J, Fu X, Hazen SL, Heijnen HFG, Dennehy MK, Liebler DC, Rader DJ, Ischiropoulos H. Increased protein nitration burden in the atherosclerotic lesions and plasma of apolipoprotein A-I-deficient mice. *Circ Res*. Published online 2007. doi:10.1161/CIRCRESAHA.107.157537

19. Watanabe J, Grijalva V, Hama S, Barbour K, Berger FG, Navab M, Fogelman AM, Reddy ST. Hemoglobin and its scavenger protein haptoglobin associate with ApoA-1-containing particles and influence the inflammatory properties and function of high density lipoprotein. *J Biol Chem*. Published online 2009. doi:10.1074/jbc.M109.017202

20. Baldo G, Fellin R, Manzatoa E, Baiocchi MR, Ongaro G, Baggio G, Fabiani F, Pauluzzi S, Crepaldi G. Characterization of hyperlipidemia m two patients with analbuminemia. *Clin Chim Acta*. Published online 1983. doi:10.1016/0009-8981(83)90330-3

21. Heinecke JW. The HDL proteome: A marker-and perhaps mediator-of coronary artery disease. *J Lipid Res*. Published online 2009. doi:10.1194/jlr.R800097-JLR200

22. Lu M, Lu Q, Zhang Y, Tian G. ApoB/apoA1 is an effective predictor of coronary heart disease risk in overweight and obesity. *J Biomed Res*. 2011;25(4):266-273. doi:10.1016/S1674-8301(11)60036-5

23. Wang Y, Tang W, Yang P, Shin H, Li Q. Hepatic NPC1L1 promotes hyperlipidemia in LDL receptor deficient mice. *Biochem Biophys Res Commun*. Published online 2018. doi:10.1016/j.bbrc.2018.03.200

24. Murphy AJ, Akhtari M, Tolani S, Pagler T, Bijl N, Kuo CL, Wang M, Sanson M, Abramowicz S, Welch C, Bochem AE, Kuivenhoven JA, Yvan-Charvet L, Tall AR. ApoE regulates hematopoietic stem cell proliferation, monocytosis, and monocyte accumulation in atherosclerotic lesions in mice. *J Clin Invest*. Published online 2011. doi:10.1172/JCI57559

25. Maneerat Y, Prasongsukarn K, Benjathummarak S, Dechkhajorn W. PPBP and DEFA1/DEFA3 genes in hyperlipidaemia as feasible synergistic inflammatory biomarkers for coronary heart disease. *Lipids Health Dis*. 2017;16(1):1-12. doi:10.1186/s12944-017-0471-0

26. Yang RZ, Lee MJ, Hu H, Pollin TI, Ryan AS, Nicklas BJ, Snitker S, Horenstein RB, Hull K, Goldberg NH, Goldberg AP, Shuldiner AR, Fried SK, Gong DW. Acute-phase serum amyloid A: An inflammatory adipokine and potential link between obesity and its metabolic complications. *PLoS Med*. Published online 2006. doi:10.1371/journal.pmed.0030287

27. Lewis KE, Kirk EA, McDonald TO, Wang S, Wight TN, O’Brien KD, Chait A. Increase in serum amyloid A evoked by dietary cholesterol is associated with increased atherosclerosis in mice. *Circulation*. Published online 2004. doi:10.1161/01.CIR.0000136819.93989.E1

28. Ridker PM, Hennekens CH, Buring JE, Rifai N. C-reactive protein and other markers of inflammation in the prediction of cardiovascular disease in women. *N Engl J Med*. Published online 2000. doi:10.1056/NEJM200003233421202

29. Zhang P, Gao J, Pu C, Feng G, Wang L, Huang L, Zhang Y. ApoM/HDL-C and apoM/apoA-I ratios are indicators of diabetic nephropathy in healthy controls and type 2 diabetes mellitus. *Clin Chim Acta*. Published online 2017. doi:10.1016/j.cca.2017.01.006

30. Zhang K, Zhang S, Zheng K, Hou Y, Liao L, He Y, Zhang L, Nebert DW, Shi J, Su Z, Xiao C. Novel P143L polymorphism of the LCAT gene is associated with dyslipidemia in Chinese patients who have coronary atherosclerotic heart disease. *Biochem Biophys Res Commun*. Published online 2004. doi:10.1016/j.bbrc.2004.03.177

31. Kappelle PJWH, de Boer JF, Perton FG, Annema W, de Vries R, Dullaart RPF, Tietge UJF. Increased LCAT activity and hyperglycaemia decrease the antioxidative functionality of HDL. *Eur J Clin Invest*. Published online 2012. doi:10.1111/j.1365-2362.2011.02604.x

32. Yan H, Fei N, Wu G, Zhang C, Zhao L, Zhang M. Regulated inflammation and lipid metabolism in colon mRNA expressions of obese germfree mice responding to Enterobacter cloacae B29 combined with the high fat diet. *Front Microbiol*. Published online 2016. doi:10.3389/fmicb.2016.01786

33. Xie C, Kang J, Burris R, Ferguson ME, Schauss AG, Nagarajan S, Wu X. Açaí juice attenuates atherosclerosis in ApoE deficient mice through antioxidant and anti-inflammatory activities. *Atherosclerosis*. Published online 2011. doi:10.1016/j.atherosclerosis.2011.02.035

34. van Himbergen TM, van Tits LJH, ter Avest E, Roest M, Voorbij HAM, de Graaf J, Stalenhoef AFH. Paraoxonase (PON1) is associated with familial combined hyperlipidemia. *Atherosclerosis*. Published online 2008. doi:10.1016/j.atherosclerosis.2007.10.017

35. Mackness B, Hunt R, Durrington PN, Mackness MI. Increased immunolocalization of paraoxonase, clusterin, and apolipoprotein A-I in the human artery wall with the progression of atherosclerosis. *Arterioscler Thromb Vasc Biol*. Published online 1997. doi:10.1161/01.ATV.17.7.1233

36. Hu T, Lin M, Zhang D, Li M, Zhang J. A UPLC/MS/MS method for comprehensive profiling and quantification of fatty acid esters of hydroxy fatty acids in white adipose tissue. *Anal Bioanal Chem*. Published online 2018. doi:10.1007/s00216-018-1350-x
