# Supplementary material for: Lipoprotein proteome profile: novel insight into hyperlipidemia
Source: Clin Transl Med. 2021 Apr 8;11(4):e361. doi: 10.1002/ctm2.361 (PMC8032137; doi:10.1002/ctm2.361)
Supplement: Supplementary file 7 — SUPPORTING INFORMATION [file CTM2-11-e361-s003.pdf]

Biological Functions	Both (73)	Human (61)	Golden Hamster (84)
**1. Lipid metabolism**	**18**	**5**	**11**
1.1 Lipid transport	**(12): APOA2, APOA4, APOB, APOC1, APOA1, APOC3, APOE, APOH, APOM, CETP,** APOC2, APOC4	**(4):** APOA5, APOD, APOF, APOL1	
1.2 Lipid Metabolism	**(6): LCAT, PLTP, AZGP1, ALB,** LDLR, RBP4	**(1):** LPA	**(11):** DBI, BPIFA2, PSAP, ANGPTL8, SNCA, SERPINA6, PCSK9, SCGB1A1, POSTN, ITGA2, GPLD1
**2. Immunity**	**33**	**28**	**23**
2.1 Complement activation	**(5): C3, CFB, CFD, CLU, IGHM**	**(4):** C4B, C9, CFHR4, C4BPA	**(9):** C2, C4, CFH, CFI, CFP, ITGAM, ITGB2,CFP, CFHRP
2.2 Inflammatory response	**(14): AHSG, HP, ORM1, PPBP, SAA1,** SAA2, SERPINA1, SERPINC1, **SERPINF2, SERPING1,** THBS1, FN1, ITIH4, SERPINF1	**(6):** AGT, IGFBP4, ORM2, PARK7, PF4, PLA2G7	**(5):** APCS, ICAM1, VCAM1, SAA3, SAA5
2.3 Immune Response	**(14):** A1BG, ACTB, **AMBP,** B2M, CAMP, CRP, **CST3,** GSN, **HPX,** KRT1, PPIA, **TF, TTR, VTN**	**(18):** CD5L, CHGA, CNN2, COL1A1, IGHA1, IGHG1, IGHG2, IGHG3, IGHV3-74, IGKC, IGLC2, JCHAIN, LBP, DEFA1, LRG1, NAPRT, UBB, SERPINA3	**(9):** CRIP1, CST6, DSC1,IHH, KRT6A, MB, POMC, RPS27A, TREML1
**3. Coagulation**	**9**	**2**	**9**
	**(9): A2M, CFL1, F2, FGA, HBB, KNG1,** PLG, SERPIND1, F5	**(2):** FBLN1, FLNA	**(9):** F13A1, FGB, FGG, HRG, ITGB1, ITIH3, KLKB1, TFPI, TMSB4X
**4. Redox activity**	**3**		**4**
	**(3): PCYOX1, PON1, GPX3**		**(4):** PRDX2, BLVRB, CP, TRX
**5. Others**	**10**	**26**	**38**
	**(10):** ANG, ARHGDIB, **HBA,** ITIH2, **KRT2, PFN1, SFTPB,** TXN, **VTDB,** KRT10	**(26):** AHNAK, BASP1, CCDC40, CSRP1, FERMT3, HPR, IGFBP2, IGFBP3, KRT33B, KRT83, KRT9, MENT, MTPN, OTOF, PDLIM1, PON3, POTEI, RNASE4, SAA2-SAA4, SH3BGRL3, SYNE1, TAGLN2, TGOLN2, TLN1, TPM4, UBTF	**(37):** AFM, ANTXR1, ARHGDIB, CA1, TNA, CNDP1, CST6, EIF5A, FAM171B, FETUB, GC, HBA, HBZ, HPP, ITIH1, ITIH4, and so on.
